# Supplementary material for: Covid19Vaxplorer: A free, online, user-friendly COVID-19 vaccine allocation comparison tool
Source: PLOS Glob Public Health. 2024 Jan 22;4(1):e0002136. doi: 10.1371/journal.pgph.0002136 (PMC10802966; doi:10.1371/journal.pgph.0002136)
Supplement: S1 Text — (PDF) [file pgph.0002136.s001.pdf]

Supporting information for **Covid19Vaxplorer**: a free, online, user-friendly COVID-19 Vaccine Allocation Comparison Tool

Imelda Trejo[1,☯], Pei-Yao Hung[2], Laura Matrajt[1,3,☯*]

1 Vaccine and Infectious Disease Division, Fred Hutchinson Cancer Center, Seattle, Washington, United States of America
2 Institute For Social Research, University of Michigan, Ann Arbor, Michigan, United States of America
3 Department of Applied Mathematics, University of Washington, Seattle, Washington, United States of America

☯These authors contributed equally to this work.
* laurama@fredhutch.org

Model Equations
Unvaccinated:

$$\begin{aligned}
\frac{dS_i}{dt} &= -\lambda_{S_i}(t) - \sum_{j=1}^{3} \theta_{S_i toSV_{ij}}(t) \\
\frac{dE_i}{dt} &= \lambda_{S_i}(t) - \gamma_E E_i \\
\frac{dA_i}{dt} &= (1-k_i)\gamma_E E_i - \gamma_A A_i \\
\frac{dP_i}{dt} &= k_i \gamma_E E_i - \gamma_P P_i \\
\frac{dI_i}{dt} &= \gamma_P P_i - (1-h_i)\gamma_I I_i - h_i \sigma I_i \\
\frac{dH_i}{dt} &= h_i \sigma I_i - \gamma_H H_i \\
\frac{dR_i}{dt} &= (1-h_i)\gamma_I I_i - 2\gamma_R R_i - \sum_{j=1}^{3} \theta_{R_i toRV_{ij}}(t) \\
\frac{dRA_i}{dt} &= \gamma_A A_i - 2\gamma_{RA} RA_i - \sum_{j=1}^{3} \theta_{RA_i toRAV_{ij}}(t) \\
\frac{dRH_i}{dt} &= \gamma_H H_i - 2\gamma_{RH} RH_i - \sum_{j=1}^{3} \theta_{RH_i toRHV_{ij}}(t) \\
\frac{dRR_i}{dt} &= 2\gamma_R R_i - 2\gamma_R RR_i - \sum_{j=1}^{3} \theta_{RR_i toRRV_{ij}}(t) \\
\frac{dRRA_i}{dt} &= 2\gamma_{RA} RA_i - 2\gamma_{RA} RRA_i - \sum_{j=1}^{3} \theta_{RRA_i toRRAV_{ij}}(t) \\
\frac{dRRH_i}{dt} &= 2\gamma_{RH} RH_i - 2\gamma_{RH} RRH_i - \sum_{j=1}^{3} \theta_{RRH_i toRRHV_{ij}}(t)
\end{aligned} \tag{1}$$

Unvaccinated with partial protection acquired from at least one infection:

$$
\begin{aligned}
\frac{dS_{P_i}}{dt} &= 2\gamma_R RR_i + 2\gamma_{RA} RRA_i + 2\gamma_{RH} RRH_i + 2\gamma_{RP} RR_{P_i} + 2\gamma_{RAP} RRA_{P_i} \\
&+ 2\gamma_{RHP} RRH_{P_i} - (1 - VE_{SUSP})\lambda_{S_{P_i}}(t) - \sum_{j=1}^{3} \theta_{SP_i toSV_{ij}}(t) \\
\frac{dE_{P_i}}{dt} &= (1 - VE_{SUSP})\lambda_{S_{P_i}}(t) - \gamma_E E_{P_i} \\
\frac{dA_{P_i}}{dt} &= (1 - (1 - VE_{SYMPP})k_i)\gamma_E E_{P_i} - \gamma_A A_{P_i} \\
\frac{dP_{P_i}}{dt} &= (1 - VE_{SYMPP})k_i \gamma_E E_{P_i} - \gamma_P P_{P_i} \\
\frac{dI_{P_i}}{dt} &= \gamma_P P_{P_i} - (1 - (1 - VE_{HP})h_i)\gamma_I I_{P_i} - (1 - VE_{HP})h_i \sigma I_{P_i} \\
\frac{dH_{P_i}}{dt} &= (1 - VE_{HP})h_i \sigma I_{P_i} - \gamma_H H_{P_i} \\
\frac{dR_{P_i}}{dt} &= (1 - (1 - VE_{HP})h_i)\gamma_I I_{P_i} - 2\gamma_{RP} R_{P_i} - \sum_{j=1}^{3} \theta_{RP_i toRB_{ij}}(t) \\
\frac{dRA_{P_i}}{dt} &= \gamma_A A_{P_i} - 2\gamma_{RAP} RA_{P_i} - \sum_{j=1}^{3} \theta_{RAP_i toRAB_{ij}}(t) \\
\frac{dRH_{P_i}}{dt} &= \gamma_H H_{P_i} - 2\gamma_{RHP} RH_{P_i} - \sum_{j=1}^{3} \theta_{RHP_i toRHB_{ij}}(t) \\
\frac{dRR_{P_i}}{dt} &= 2\gamma_{RP} R_{P_i} - 2\gamma_{RP} RR_{P_i} - \sum_{j=1}^{3} \theta_{RRP_i toRRB_{ij}}(t) \\
\frac{dRRA_{P_i}}{dt} &= 2\gamma_{RAP} RA_{P_i} - 2\gamma_{RAP} RRA_{P_i} - \sum_{j=1}^{3} \theta_{RRAP_i toRRAB_{ij}}(t) \\
\frac{dRRH_{P_i}}{dt} &= 2\gamma_{RHP} RH_{P_i} - 2\gamma_{RHP} RRH_{P_i} - \sum_{j=1}^{3} \theta_{RRHP_i toRRHB_{ij}}(t)
\end{aligned}
\tag{2}
$$

Vaccinated with primary series, without distinguishing the vaccine products, and with partial protection provided from vaccines and infection:

$$
\begin{aligned}
\frac{dS_{W_i}}{dt} &= 2\gamma_{RW} RR_{W_i} + 2\gamma_{RAW} RRA_{W_i} + 2\gamma_{RHW} RRH_{W_i} + \sum_{j=1}^{3} \gamma_{SV_j} S_{V_{ij}} \\
&\quad - (1 - VE_{SUSW})\lambda_{S_{W_i}}(t) - \sum_{k=1}^{3}\sum_{j=1}^{3} \theta_{SW_{i,V_k} toSB_{ij}}(t) \\
\frac{dE_{W_i}}{dt} &= (1 - VE_{SUSW})\lambda_{S_{W_i}}(t) - \gamma_E E_{W_i} \\
\frac{dA_{W_i}}{dt} &= (1 - (1 - VE_{SYMPW})k_i)\gamma_E E_{W_i} - \gamma_A A_{W_i} \\
\frac{dP_{W_i}}{dt} &= (1 - VE_{SYMPW})k_i \gamma_E E_{W_i} - \gamma_P P_{W_i} \\
\frac{dI_{W_i}}{dt} &= \gamma_P P_{W_i} - (1 - (1 - VE_{HW})h_i)\gamma_I I_{W_i} - (1 - VE_{HW})h_i \sigma I_{W_i} \\
\frac{dH_{W_i}}{dt} &= (1 - VE_{HW})h_i \sigma I_{W_i} - \gamma_H H_{W_i} \\
\frac{dR_{W_i}}{dt} &= (1 - (1 - VE_{HW})h_i)\gamma_I I_{W_i} - 2\gamma_{RW} R_{W_i} - \sum_{k=1}^{3}\sum_{j=1}^{3} \theta_{RW_{i,V_k} toRB_{ij}}(t) \\
\frac{dRA_{W_i}}{dt} &= \gamma_A A_{W_i} - 2\gamma_{RAW} RA_{W_i} - \sum_{k=1}^{3}\sum_{j=1}^{3} \theta_{RAW_{i,V_k} toRAB_{ij}}(t) \\
\frac{dRH_{W_i}}{dt} &= \gamma_H H_{W_i} - 2\gamma_{RHW} RH_{W_i} - \sum_{k=1}^{3}\sum_{j=1}^{3} \theta_{RHW_{i,V_k} toRHB_{ij}}(t) \\
\frac{dRR_{W_i}}{dt} &= 2\gamma_{RW} R_{W_i} - 2\gamma_{RW} RR_{W_i} - \sum_{k=1}^{3}\sum_{j=1}^{3} \theta_{RRW_{i,V_k} toRRB_{ij}}(t) \\
\frac{dRRA_{W_i}}{dt} &= 2\gamma_{RAW} RA_{W_i} - 2\gamma_{RAW} RRA_{W_i} - \sum_{k=1}^{3}\sum_{j=1}^{3} \theta_{RRAW_{i,V_k} toRRAB_{ij}}(t) \\
\frac{dRRH_{W_i}}{dt} &= 2\gamma_{RHW} RH_{W_i} - 2\gamma_{RHW} RRH_{W_i} - \sum_{k=1}^{3}\sum_{j=1}^{3} \theta_{RRHW_{i,V_k} toRRHB_{ij}}(t)
\end{aligned} \tag{3}
$$

Vaccinated with primary series and vaccine product j:

$$\begin{aligned}
\frac{dS_{V_{ij}}}{dt} &= \theta_{S_i toSV_{ij}}(t) + \theta_{SP_i toSV_{ij}}(t) + \gamma_{SB_j} S_{B_{ij}} - \sum_{k=1}^{3} \theta_{SV_{ij} toSB_{ik}}(t) \\
&\quad - (1 - VE_{SUSV_j})\lambda_{S_{V_{ij}}}(t) - \gamma_{SV_j} S_{V_{ij}} \\
\frac{dE_{V_{ij}}}{dt} &= (1 - VE_{SUSV_j})\lambda_{S_{V_{ij}}}(t) - \gamma_E E_{V_{ij}} \\
\frac{dA_{V_{ij}}}{dt} &= (1 - (1 - VE_{SYMPV_j})k_i)\gamma_E E_{V_{ij}} - \gamma_A A_{V_{ij}} \\
\frac{dP_{V_{ij}}}{dt} &= (1 - VE_{SYMPV_j})k_i \gamma_E E_{V_{ij}} - \gamma_P P_{V_{ij}} \\
\frac{dI_{V_{ij}}}{dt} &= \gamma_P P_{V_{ij}} - (1 - (1 - VE_{HV_j})h_i)\gamma_I I_{V_{ij}} - (1 - VE_{HV_j})h_i \sigma I_{V_{ij}} \\
\frac{dH_{V_{ij}}}{dt} &= (1 - VE_{HV_j})h_i \sigma I_{V_{ij}} - \gamma_H H_{V_{ij}} \\
\frac{dR_{V_{ij}}}{dt} &= (1 - (1 - VE_{HV_j})h_i)\gamma_I I_{V_{ij}} - 2\gamma_{RV_j} R_{V_{ij}} - \sum_{k=1}^{3} \theta_{RV_{ij} toRB_{ik}}(t) \\
\frac{dRA_{V_{ij}}}{dt} &= \gamma_A A_{V_{ij}} - 2\gamma_{RAV_{ij}} RA_{V_{ij}} - \sum_{k=1}^{3} \theta_{RAV_{ij} toRAB_{ik}}(t) \\
\frac{dRH_{V_{ij}}}{dt} &= \gamma_H H_{V_{ij}} - 2\gamma_{RHV_{ij}} RH_{V_{ij}} - \sum_{k=1}^{3} \theta_{RHV_{ij} toRHB_{ik}}(t) \\
\frac{dRR_{V_{ij}}}{dt} &= 2\gamma_{RV_j} R_{V_{ij}} - 2\gamma_{RV_j} RR_{V_{ij}} - \sum_{k=1}^{3} \theta_{RRV_{ij} toRRB_{ik}}(t) \\
\frac{dRRA_{V_{ij}}}{dt} &= 2\gamma_{RAV_j} RA_{V_{ij}} - 2\gamma_{RAV_j} RRA_{V_{ij}} - \sum_{k=1}^{3} \theta_{RRAV_{ij} toRRAB_{ik}}(t) \\
\frac{dRRH_{V_{ij}}}{dt} &= 2\gamma_{RHV_j} RH_{V_{ij}} - 2\gamma_{RHV_j} RRH_{V_{ij}} - \sum_{k=1}^{3} \theta_{RRHV_{ij} toRRHB_{ik}}(t)
\end{aligned} \tag{4}$$

Vaccinated and boosted with vaccine product j:

$$
\begin{aligned}
\frac{dS_{B_{ij}}}{dt} &= \sum_{k=1}^{3} \theta_{SV_{ik}toSB_{ij}}(t) + \sum_{k=1}^{3} \theta_{SW_{i,V_k}toSB_{ij}}(t) + 2\gamma_{RV_j} RR_{V_{ij}} + 2\gamma_{RAV_j} RRA_{V_{ij}} \\
&+ 2\gamma_{RHV_j} RRH_{V_{ij}} + 2\gamma_{RB_j} RR_{B_{ij}} + 2\gamma_{RAB_j} RRA_{B_{ij}} + 2\gamma_{RHB_j} RRH_{B_{ij}} \\
&- (1 - VE_{SUSB_j})\lambda_{S_{B_{ij}}}(t) - \gamma_{SB_j} S_{B_{ij}} \\
\frac{dE_{B_{ij}}}{dt} &= (1 - VE_{SUSB_j})\lambda_{S_{B_{ij}}}(t) - \gamma_E E_{B_{ij}} \\
\frac{dA_{B_{ij}}}{dt} &= (1 - (1 - VE_{SYMPB_j}))k_i \gamma_E E_{B_{ij}} - \gamma_A A_{B_{ij}} \\
\frac{dP_{B_{ij}}}{dt} &= (1 - VE_{SYMPB_j})k_i \gamma_E E_{B_{ij}} - \gamma_P P_{B_{ij}} \\
\frac{dI_{B_{ij}}}{dt} &= \gamma_P P_{B_{ij}} - (1 - (1 - VE_{HB_j}))h_i \gamma_I I_{B_{ij}} - (1 - VE_{HB_j})h_i \sigma I_{B_{ij}} \\
\frac{dH_{B_{ij}}}{dt} &= (1 - VE_{HB_j})h_i \sigma I_{B_{ij}} - \gamma_H H_{B_{ij}} \\
\frac{dR_{B_{ij}}}{dt} &= (1 - (1 - VE_{HB_j}))h_i \gamma_I I_{B_{ij}} + \theta_{RP_i toRB_{ij}}(t) + \sum_{k=1}^{3} \theta_{RW_{i,V_k}toRB_{ij}}(t) \\
&+ \sum_{k=1}^{3} \theta_{RV_{ik}toRB_{ij}}(t) - 2\gamma_{RB_j} R_{B_{ij}} \\
\frac{dRA_{B_{ij}}}{dt} &= \gamma_A A_{B_{ij}} + \theta_{RAP_i toRAB_{ij}}(t) + \sum_{k=1}^{3} \theta_{RAW_{i,V_k}toRAB_{ij}}(t) + \sum_{k=1}^{3} \theta_{RAV_{ik}toRAB_{ij}}(t) \\
&- 2\gamma_{RAB_j} RA_{B_{ij}} \\
\frac{dRH_{B_{ij}}}{dt} &= \gamma_H H_{B_{ij}} + \theta_{RHP_i toRHB_{ij}}(t) + \sum_{k=1}^{3} \theta_{RHW_{i,V_k}toRHB_{ij}}(t) + \sum_{k}^{3} \theta_{RHV_{ik}toRHB_{ij}}(t) \\
&- 2\gamma_{RHB_j} RH_{B_{ij}} \\
\frac{dRR_{B_{ij}}}{dt} &= 2\gamma_{RB_j} R_{B_{ij}} + \theta_{RRP_i toRRB_{ij}}(t) + \sum_{k=1}^{3} \theta_{RRW_{i,V_k}toRRB_{ij}}(t) + \sum_{k=1}^{3} \theta_{RRV_{ik}toRRB_{ij}}(t) \\
&- 2\gamma_{RB_j} RR_{B_{ij}} \\
\frac{dRRA_{B_{ij}}}{dt} &= 2\gamma_{RAB_j} RA_{B_{ij}} + \theta_{RRAP_i toRRAB_{ij}}(t) + \sum_{k=1}^{3} \theta_{RRAW_{i,V_k}toRRAB_{ij}}(t) \\
&+ \sum_{k=1}^{3} \theta_{RRAV_{ik}toRRAB_{ij}}(t) - 2\gamma_{RA_{B_j}} RRA_{B_{ij}} \\
\frac{dRRH_{B_{ij}}}{dt} &= 2\gamma_{RH_{B_j}} RH_{B_{ij}} + \theta_{RRHP_i toRRHB_{ij}}(t) + \sum_{k=1}^{3} \theta_{RRHW_{i,V_k}toRRHB_{ij}}(t) \\
&+ \sum_{k=1}^{3} \theta_{RRHV_{ik}toRRHB_{ij}}(t) - 2\gamma_{RH_{B_j}} RRH_{B_{ij}}.
\end{aligned} \tag{5}
$$

Force of infection and contact matrix. The force of infection for each compartment, $\alpha = i, P_i, W_i, V_{ij}, B_{ij}$, $i = 1, 2, \ldots, 5$ and $j = 1, 2, 3$, is:

$$\lambda_{S_\alpha}(t) = m_i \lambda_i S_\alpha,$$

where

$$\lambda_i = \beta \sum_{\alpha \in \{k, P_k, W_k, V_{kj}, B_{kj}\}_{k=1,2,\ldots,5, j=1,2,3}} \frac{C_{i,k}(r_A A_\alpha + r_P P_\alpha + r_H H_\alpha + I_\alpha)}{N_k}. \tag{6}$$

N_i and m_i are the population size and the relative susceptibility of age-group $i = 1, 2, \ldots, 5$, respectively. r_A, r_P, r_H are the relative infectiousness of asymptomatic, pre-symptomatic, and hospitalized infectious individuals. $C = d_1 M_H + d_2 M_W + d_3 M_S + d_4 M_O$ is the contact matrix defined as a linear combination of the contact matrices at home (M_H), work (M_W), school (M_S) and other locations (M_O), d_l is the percentage of social distancing, $l = 1, 2, 3, 4$. See [1] for more details about location-specific contact matrices. Matrices that we adapted into the five age-groups: 0–19, 20–49, 50–64, 65–74, and those 75 and older and are consistent, i. e., the mean number of contacts made by members of the age group i with members of the age group k at a given location is:

$$M_{ik} = \frac{1}{2N_i}(M_{ik}N_i + M_{ki}N_k),$$

$i, k = 1, 2, \ldots, 5$.

Vaccination Rate. We denote with $V_{ij}(t)$ the number of vaccines of vaccine product j to allocate per day among the available individuals $X_i(t)$ from age-group i at time $t \geq 0$, $i = 1, 2, \ldots, 5$, $j = 1, 2, 3$. The vaccination rate at time t is

$$\theta_{V_{ij}}(X_i(t)) = \theta_{X_i toXV_{ij}}(t) = \begin{cases} 0 & \text{if } t > t^* \\ \min\{X_i(t), V_{ij}(t)\} & \text{if } 0 \leq t \leq t^*, \end{cases} \tag{7}$$

where t^* is the ending time of the vaccination campaign with vaccine type j.

Rate of transmission and the basic reproduction number. The basic reproduction number is the expected number of secondary infections an infected individual will generate in a fully susceptible population. It is an important epidemic parameter that characterizes the growth of the infected population in a naïve population. It is important to note that R_0 is different from the effective reproductive number, R_{eff}, defined as the expected number of secondary infections an infected individual will produce in a partially susceptible population. Given a value of R_{eff}The user needs to make assumptions about the proportion of the population with some level of immunity to obtain an estimate for R_0. However, based on work by [2], **Covid19Vaxplorer** provides default values for R_0 for several SARS-CoV-2 strains. **Covid19Vaxplorer** takes R_0 as an input parameter and it is then used to compute β, the probability of infection given contact. We use the method developed in [3] to obtain β as follows.

The vector state of the system of equations (1), with $d_l = 1$ for all locations, is

$$x = (E_1, \ldots, E_5, A_1, \ldots, A_5, P_1, \ldots, P_5, I_1, \ldots, I_5, H_1, \ldots, H_5, S_1, \ldots, S_5, R_1, \ldots, R_5,$$

$$RA_1, \ldots, RA_5, RH_1, \ldots, RH_5, RR_1, \ldots, RR_5, RRA_1, \ldots, RRA_5, RRH_1, \ldots, RRH_5)$$

and $dx/dt = \mathcal{F}(x) - \mathcal{V}(x)$, where $\mathcal{F}$ is the rate of new infections entering the population, and $\mathcal{V}$ is the rate of movement (by other means) out of, and into, each compartment, respectively. We denote with A_0 and V_0 the Jacobian matrices of the first five block submatrices of $\mathcal{F}$ and $\mathcal{V}$, respectively, evaluated at the disease-free equilibrium:

$$x_{dfe} = (\mathbf{0}, \mathbf{0}, \mathbf{0}, \mathbf{0}, \mathbf{0}, N_1, N_2, N_3, N_4, N_5, \mathbf{0}, \mathbf{0}, \mathbf{0}, \mathbf{0}, \mathbf{0}, \mathbf{0}),$$

N_i is the total population of age-group i, $i = 1, 2, 3, 4, 5$, and $\mathbf{0} \in \mathbf{R}^5$. Then,

$$A_0 = \beta F_0 = \beta \begin{pmatrix} \mathbf{0} & r_A C_1 & r_P C_1 & C_1 & r_H C_1 \\ \mathbf{0} & \mathbf{0} & \mathbf{0} & \mathbf{0} & \mathbf{0} \\ \mathbf{0} & \mathbf{0} & \mathbf{0} & \mathbf{0} & \mathbf{0} \\ \mathbf{0} & \mathbf{0} & \mathbf{0} & \mathbf{0} & \mathbf{0} \\ \mathbf{0} & \mathbf{0} & \mathbf{0} & \mathbf{0} & \mathbf{0} \end{pmatrix}$$

and,

$$V_0 = \begin{pmatrix} \gamma_E \mathbf{I} & \mathbf{0} & \mathbf{0} & \mathbf{0} & \mathbf{0} \\ -\gamma_E \mathbf{I}(1-k) & \gamma_A \mathbf{I} & \mathbf{0} & \mathbf{0} & \mathbf{0} \\ -\gamma_E \mathbf{I} k & \mathbf{0} & \gamma_P \mathbf{I} & \mathbf{0} & \mathbf{0} \\ \mathbf{0} & \mathbf{0} & -\gamma_P \mathbf{I} & \mathbf{I}(\gamma_I(1-h) + \sigma h) & \mathbf{0} \\ \mathbf{0} & \mathbf{0} & \mathbf{0} & -\sigma \mathbf{I} h & \gamma_H \mathbf{I} \end{pmatrix},$$

where $\mathbf{I}$ is the identity matrix of dimension 5×5 and C_1 is the 5×5 matrix whose entries are $m_i C_{ij} N_i / N_j$, $i, j = 1, \ldots, 5$. The basic reproduction number is [3]:

$$\mathcal{R}_0 = \beta\, \rho(F_0 V_0^{-1}),$$

where $\rho(F_0 V_0^{-1})$ denotes the real part of the spectral radius of the matrix $F_0 V_0^{-1}$. Then, the transmission rate is

$$\beta = \frac{\mathcal{R}_0}{\rho(F_0 V_0^{-1})}.$$

Data sources and model default parameters

Population structure: We use contact matrices for regions given in [1]. The contact matrices for the following regions: Australia, Haiti, Japan, Lebanon, Somalia, Taiwan were imputed by assuming similar contacts as the neighboring countries (New Zealand, Dominican Republic, South Korea, Syria, Djibouti and South Korea respectively). For each region, the total population and age distribution were obtained from [4], with population densities correspond to the 2022 estimates.

Vaccine effectiveness: Vaccine effectiveness parameters were based on the data presented in [5]. We assume that there is a multiplicative relationship between the vaccine efficacy against laboratory-confirmed COVID-19 disease, $\mathrm{VE_{DIS}}, \mathrm{VE_{SUS}}$ and $\mathrm{VE_{SYMP}}$ [6], so that

$$\mathrm{VE_{DIS}} = 1 - (1 - \mathrm{VE_{SUS}})(1 - \mathrm{VE_{SYMP}}). \tag{8}$$

And similarly,

$$\mathrm{VE_{SEV}} = 1 - (1 - \mathrm{VE_{H}})(1 - \mathrm{VE_{SYMP}}). \tag{9}$$

Using this relationship and early vaccine effectiveness estimates, we derived $\mathrm{VE_{SYMP}}$ and $\mathrm{VE_{H}}$ from given estimates for $\mathrm{VE_{DIS}}$ and $\mathrm{VE_{SEV}}$. Furthermore, when vaccine effectiveness estimates were not available for Omicron, we imputed them in the following way: we computed the reduction in the effectiveness between ancestral strain and Omicron for those vaccine products for which the data was available, and use that as a multiplier for those products for which we could not find an estimate. Table **??** summarizes the default values for vaccine effectiveness for each vaccine product provided.

Natural history Parameters: Hospitalization and mortality rates were obtained from [7]. Because the rates in [7] are given for different age groups than the ones we considered, we adjusted them to each region's population in agreement with that region's population composition.

S1 Table provides the default parameters provided in **Covid19Vaxplorer**. However, the user can change all these parameters to match their particular location and experience.

References

1. Prem K, Zandvoort Kv, Klepac P, Eggo RM, Davies NG, for the Mathematical Modelling of Infectious Diseases COVID-19 Working Group C, et al. Projecting contact matrices in 177 geographical regions: An update and comparison with empirical data for the COVID-19 era. PLOS Computational Biology. 2021;17(7):1–19. doi:10.1371/journal.pcbi.1009098.

2. Moore S, Hill EM, Dyson L, Tildesley MJ, Keeling MJ. Retrospectively modeling the effects of increased global vaccine sharing on the COVID-19 pandemic. Nature Medicine. 2022;28(11):2416–2423. doi:10.1038/s41591-022-02064-y.

3. van den Driessche P, Watmough J. Reproduction numbers and sub-threshold endemic equilibria for compartmental models of disease transmission. Math Biosci. 2002;180:29–48. doi:10.1016/s0025-5564(02)00108-6.

4. United Nations, Department of Economic and Social Affairs Population Division. World Population Prospects: Total population (both sexes combined) by five-year age group; 2019. https://population.un.org/wpp/Download/Standard/Population/. Available from: https://population.un.org/wpp/Download/Standard/Population/ [cited April 8, 2022].
5. Barber RM, Sorensen RJD, Pigott DM, Bisignano C, Carter A, Amlag JO, et al. Estimating global, regional, and national daily and cumulative infections with SARS-CoV-2 through Nov 14, 2021: a statistical analysis. The Lancet. 2022;399(10344):2351–2380. doi:10.1016/S0140-6736(22)00484-6.
6. Mehrotra DV, Janes HE, Fleming TR, Annunziato PW, Neuzil KM, Carpp LN, et al. Clinical Endpoints for Evaluating Efficacy in COVID-19 Vaccine Trials. Annals of Internal Medicine. 2020;doi:10.7326/M20-6169.
7. Ferguson NM, Laydon D, Nedjati-Gilani G, Imai N, Ainslie K, Baguelin M, et al. Impact of non-pharmaceutical interventions (NPIs) to reduce COVID-19 mortality and healthcare demand. 2020;.
