# Supplementary figures and images for: Covid19Vaxplorer: A free, online, user-friendly COVID-19 vaccine allocation comparison tool
Source: PLOS Glob Public Health. 2024 Jan 22;4(1):e0002136. doi: 10.1371/journal.pgph.0002136 (PMC10802966; doi:10.1371/journal.pgph.0002136)

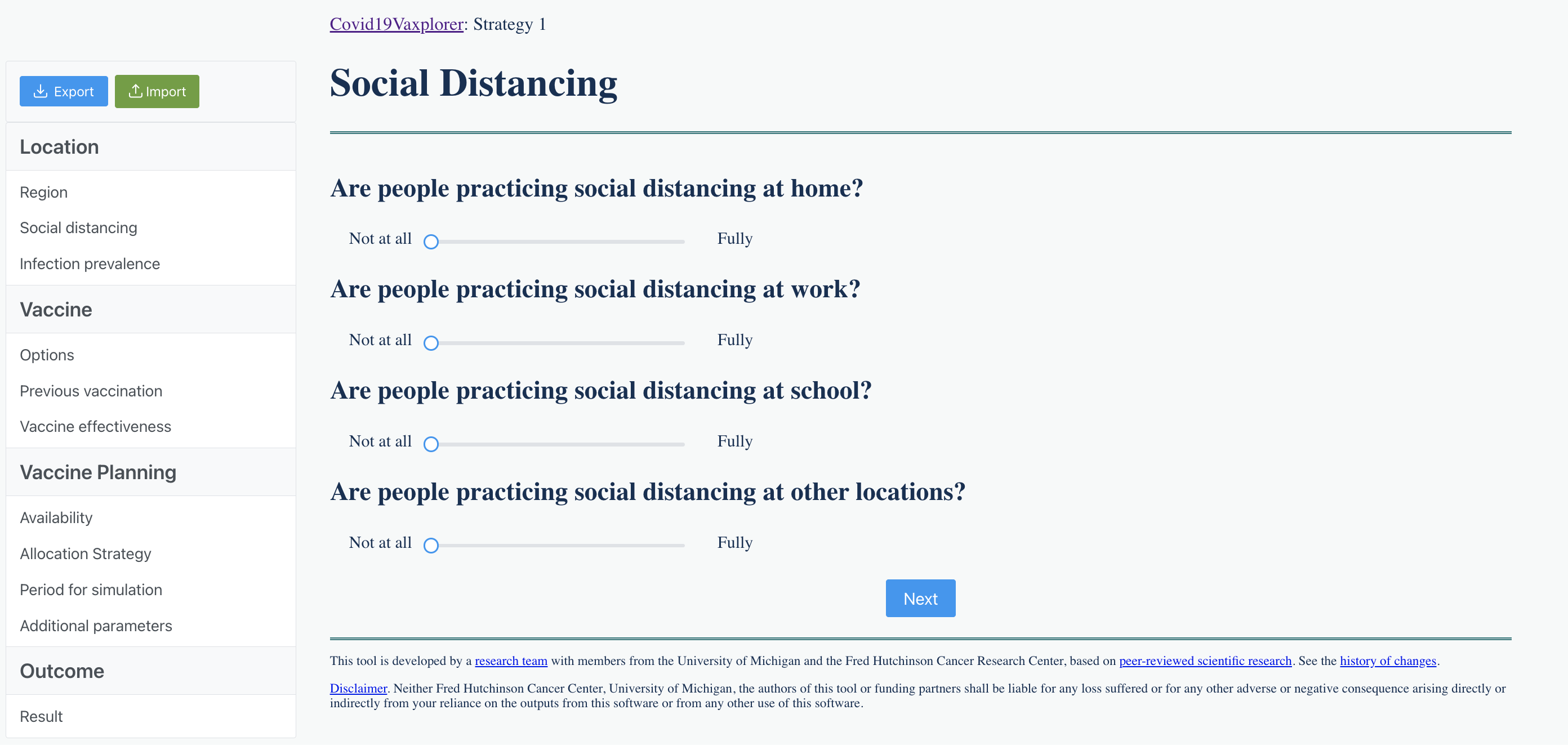

Supplement: S1 Fig — The user can select for each location a multiplier (using a sliding bar) representing the reduction in the number of contacts in that particular location. (PNG) [file pgph.0002136.s002.png]

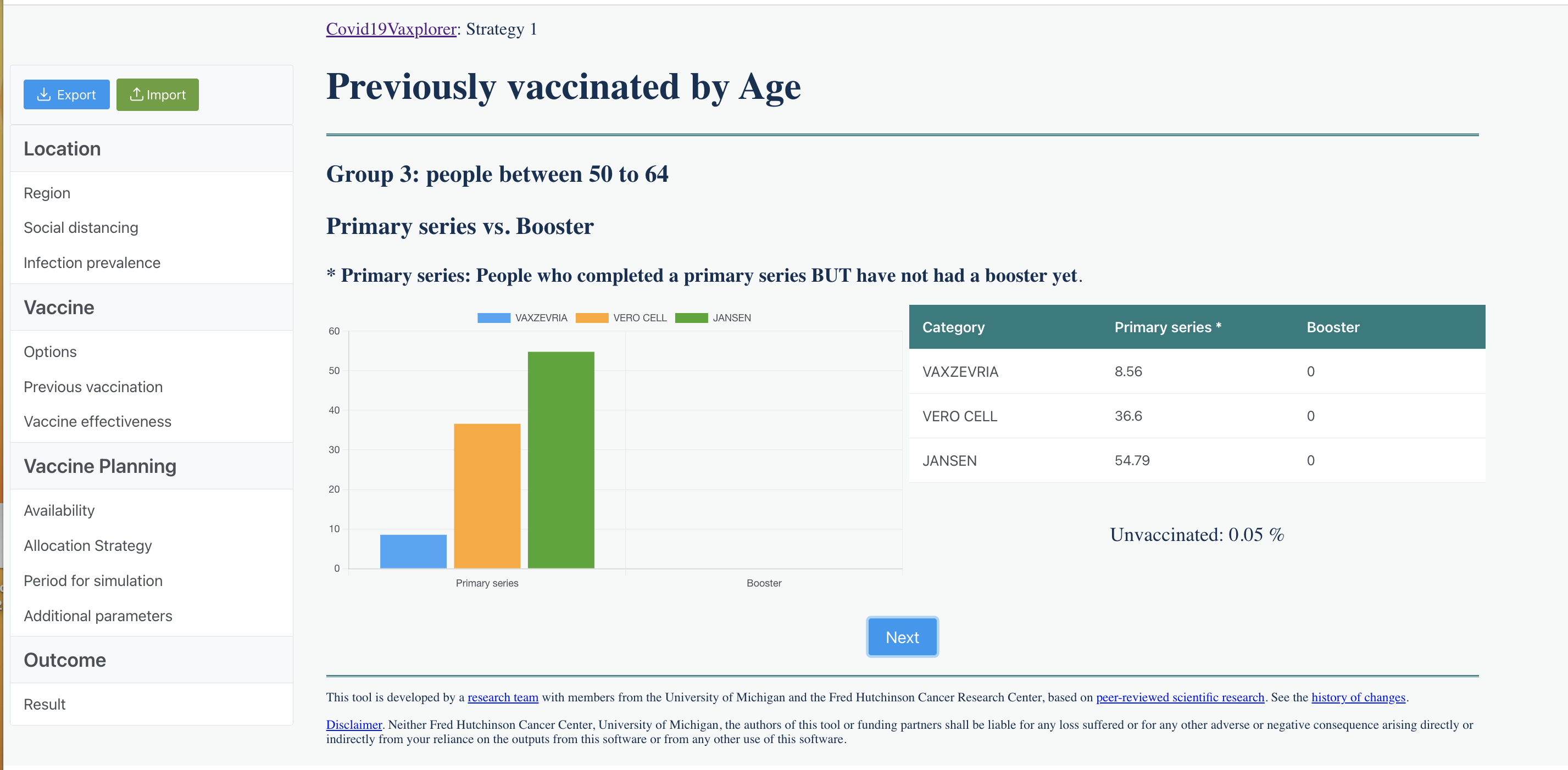

Supplement: S2 Fig — There are five such windows, one per age group (group 3 is visualized here). In each window, the user inputs the proportion of that age group that has been previously vaccinated either with a primary series or with a booster for each vaccine product. (PNG) [file pgph.0002136.s003.png]

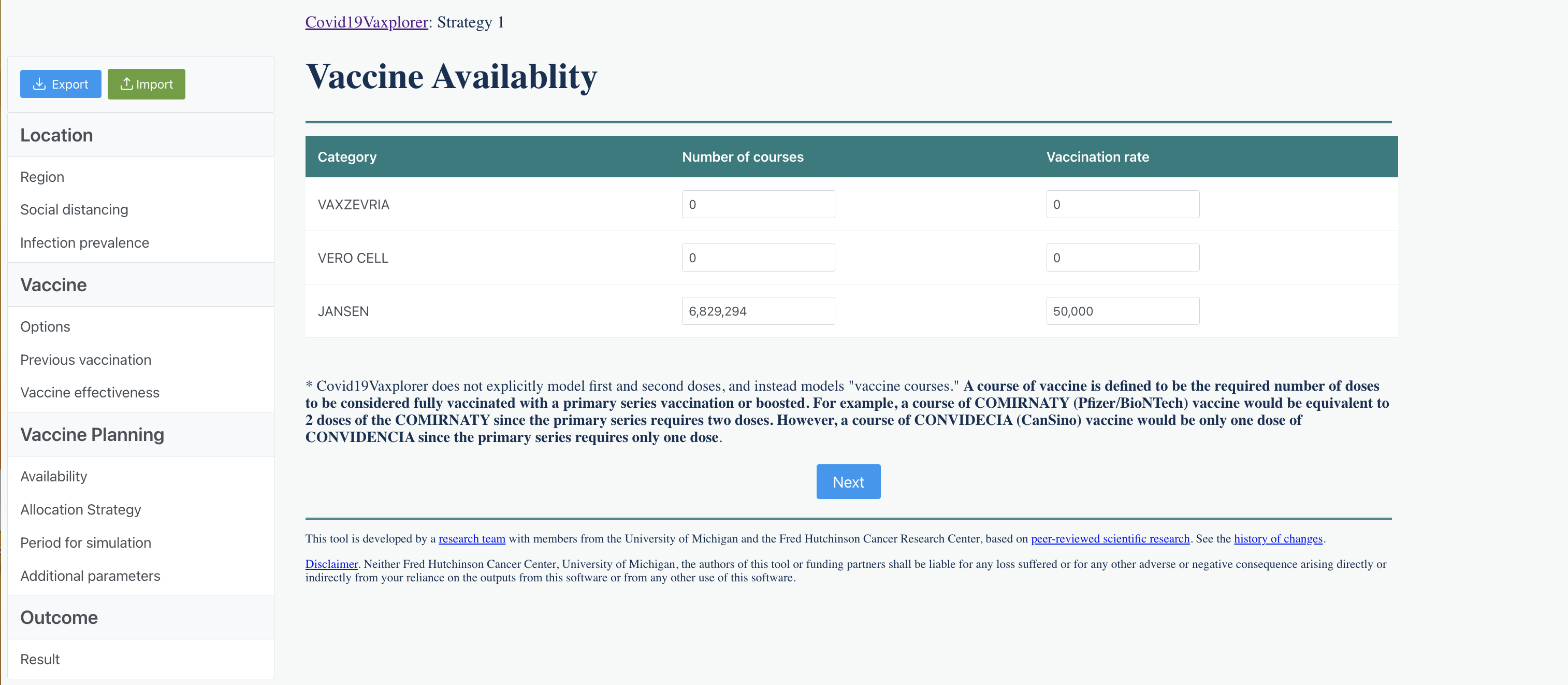

Supplement: S3 Fig — The user inputs for each vaccine product the amount of product available and the vaccination rate (i.e. the number of doses to be distributed per day). (PNG) [file pgph.0002136.s004.png]

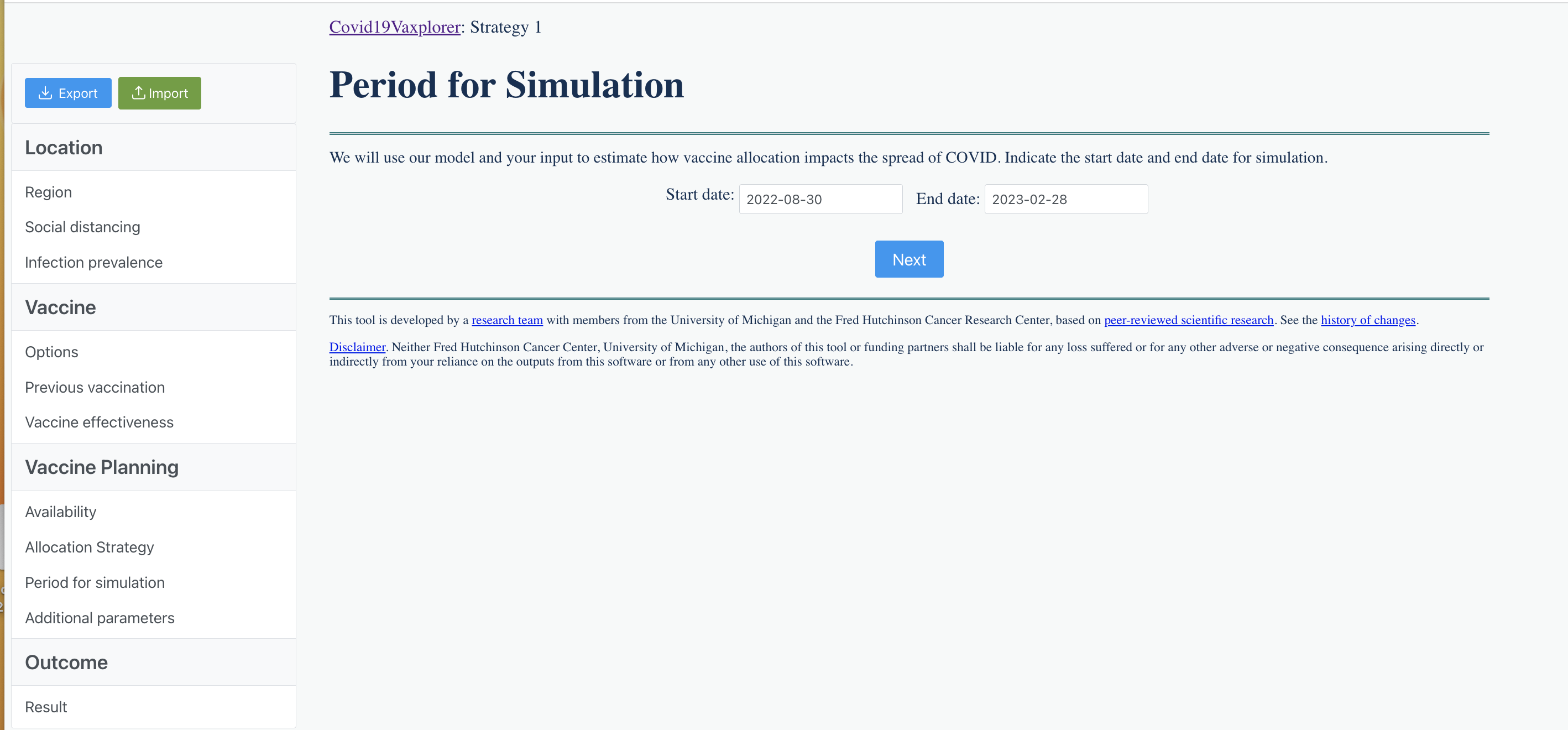

Supplement: S4 Fig — The tool provides a default end of simulation date based on the number of vaccines to be allocated and the vaccination rates (full details in Methods), but the user can change the default values. (PNG) [file pgph.0002136.s005.png]

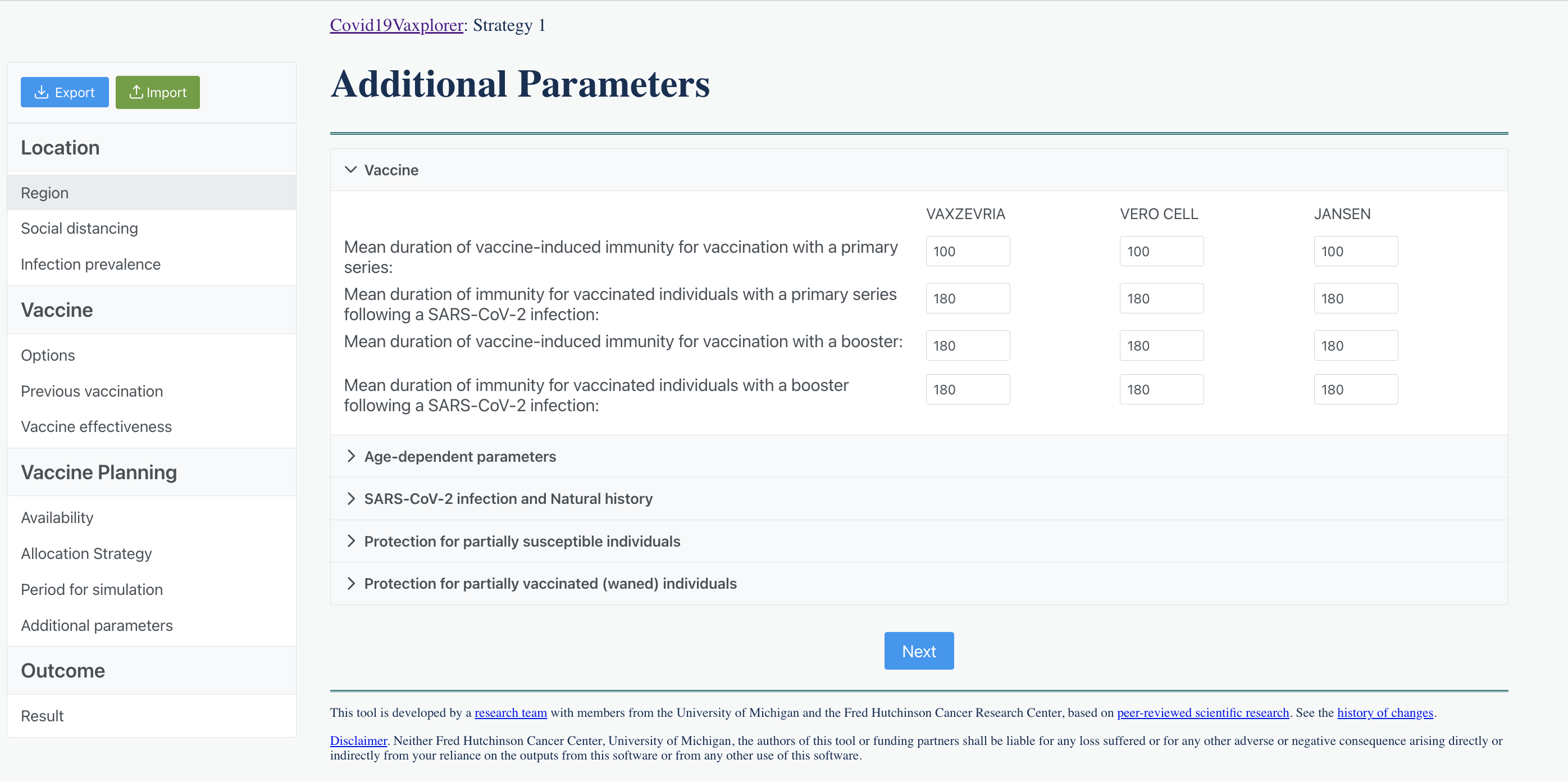

Supplement: S5 Fig — Additional parameters for the model are displayed in this window. While Covid19Vaxplorer has default parameters, the user can modify all of them. (PNG) [file pgph.0002136.s006.png]

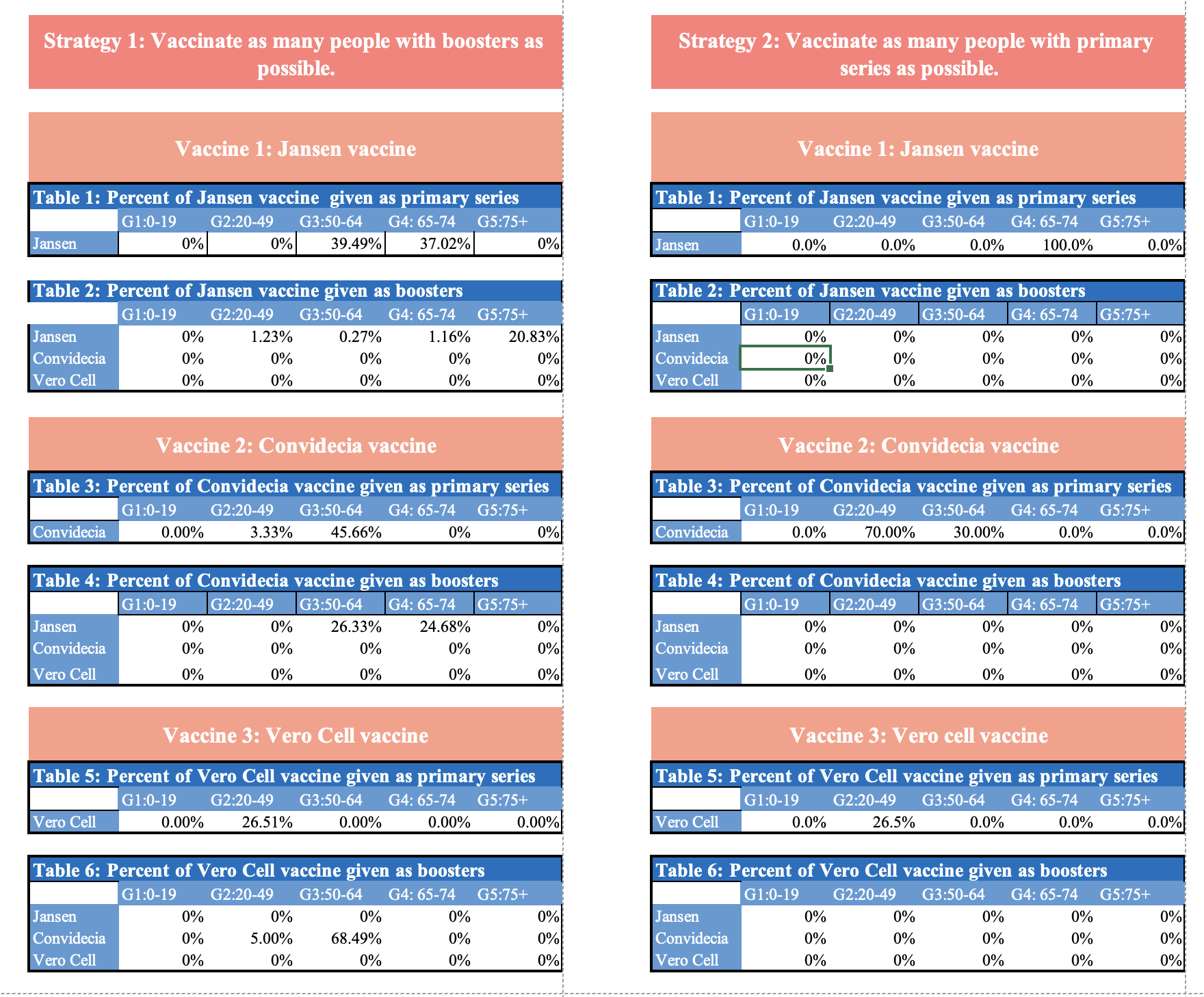

Supplement: S6 Fig — (PNG) [file pgph.0002136.s007.png]

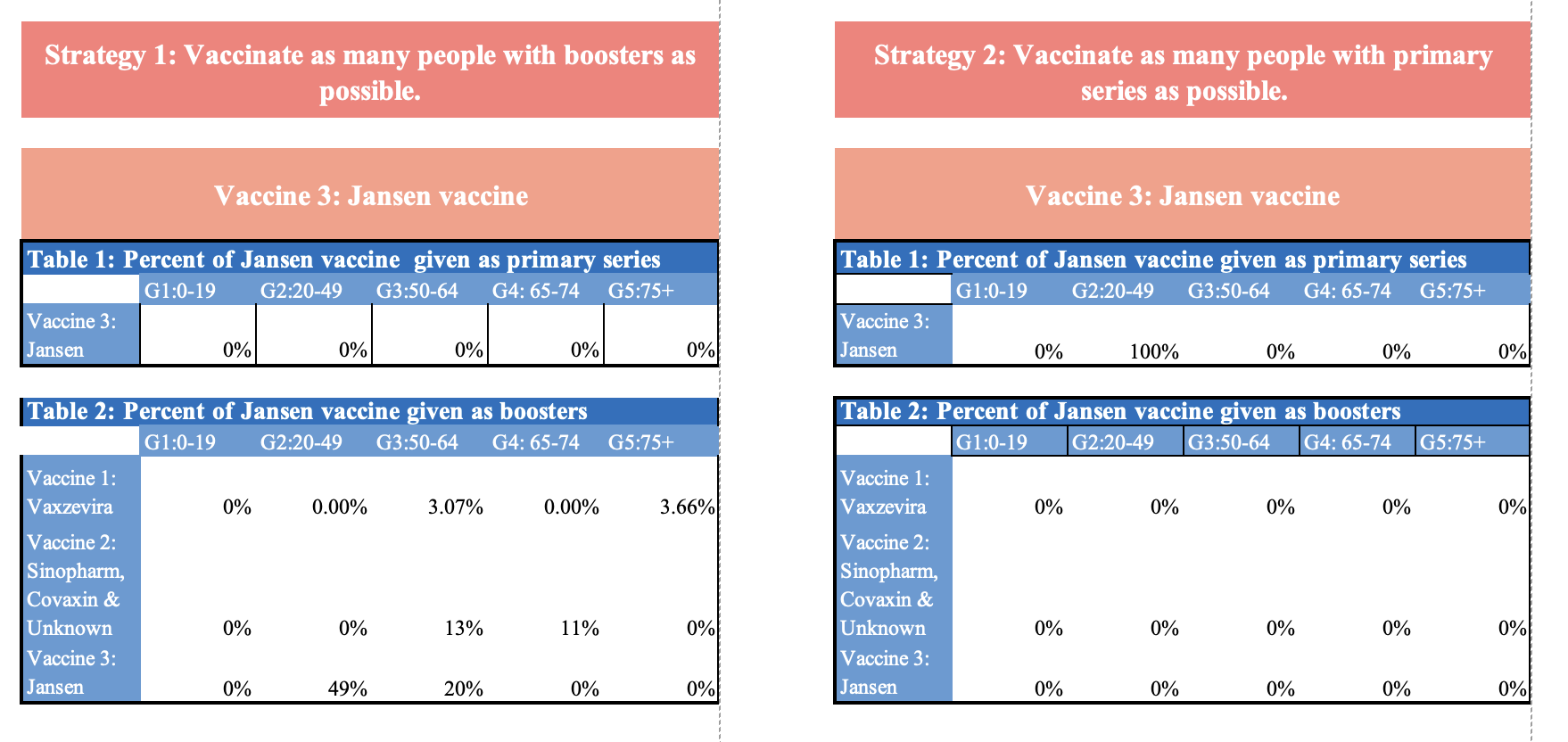

Supplement: S7 Fig — Strategy 1, “boosters first” strategy, would result in 8,836 cumulative deaths at the end of the simulation period. In contrast, Strategy 2, “primary series first” would result in 6791 cumulative deaths. (PNG) [file pgph.0002136.s008.png]
