# Supplementary material for: Covid19Vaxplorer: A free, online, user-friendly COVID-19 vaccine allocation comparison tool
Source: PLOS Glob Public Health. 2024 Jan 22;4(1):e0002136. doi: 10.1371/journal.pgph.0002136 (PMC10802966; doi:10.1371/journal.pgph.0002136)
Supplement: S8 Fig — (PDF) [file pgph.0002136.s009.pdf]

Covid19Vaxplorer

Export Import

Location

Region

Social distancing

Infection

Infection prevalence

Vaccine

Options

Previous vaccination

Vaccine effectiveness

Vaccine Planning

Availability

Allocation Strategy

Period for simulation

Additional parameters

Outcome

Result

Result

Vaccination Strategy Simulated Outcome:

Strategy 1

Region: Haiti

Population size: 11,402,533

Cumulative number of deaths over the simulation period: 8836

Maximum number of hospitalized individuals: 2728

Cumulative number of deaths:

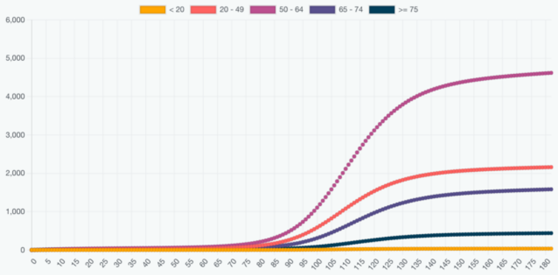

Number of hospitalized people:

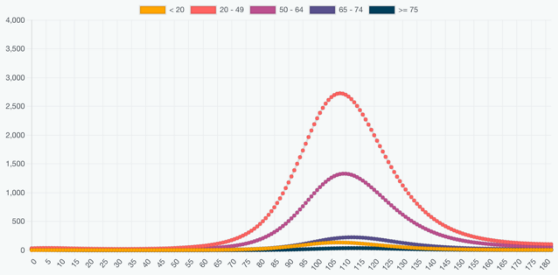

Number of symptomatic infections:

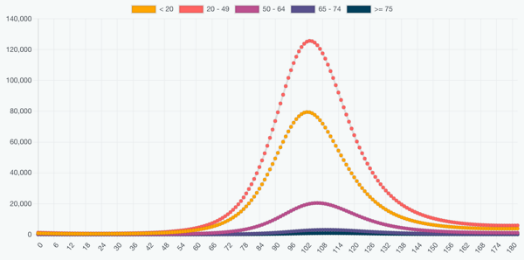

Number of infected people:

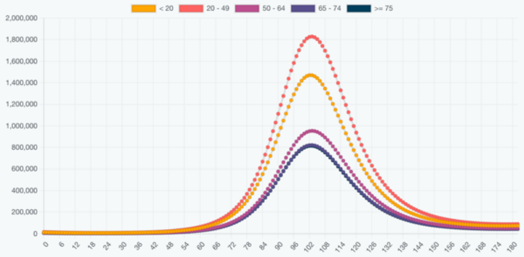

Edit Duplicate Remove Export Result

Strategy 2

Region: Haiti

Population size: 11,402,533

Cumulative number of deaths over the simulation period: 6791

Maximum number of hospitalized individuals: 2451

Cumulative number of deaths:

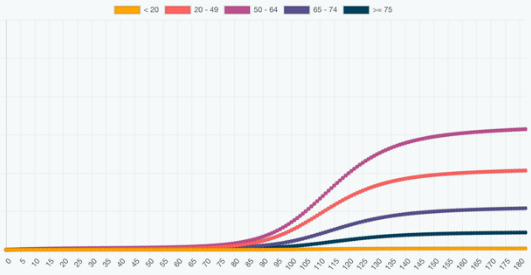

Number of hospitalized people:

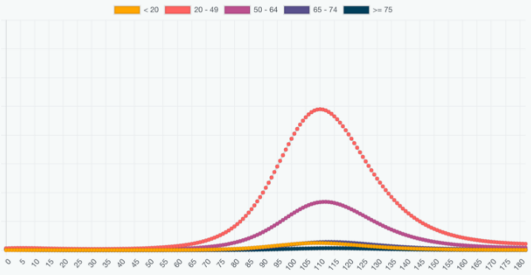

Number of symptomatic infections:

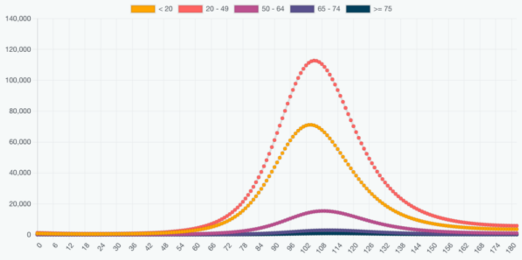

Number of infected people:

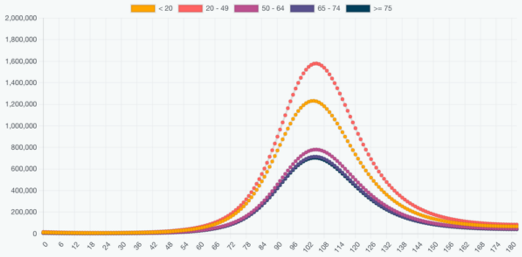

Edit Duplicate Remove Export Result

This tool is developed by a research team with members from the University of Michigan and the Fred Hutchinson Cancer Research Center, based on peer-reviewed scientific research. See the history of changes.

Disclaimer. Neither Fred Hutchinson Cancer Center, University of Michigan, the authors of this tool or funding partners shall be liable for any loss suffered or for any other adverse or negative consequence arising directly or indirectly from your reliance on the outputs from this software or from any other use of this software.
