# Supplementary material for: Covid19Vaxplorer: A free, online, user-friendly COVID-19 vaccine allocation comparison tool
Source: PLOS Glob Public Health. 2024 Jan 22;4(1):e0002136. doi: 10.1371/journal.pgph.0002136 (PMC10802966; doi:10.1371/journal.pgph.0002136)
Supplement: S1 Table — (PDF) [file pgph.0002136.s010.pdf]

| Parameter | Value | Reference |
| --- | --- | --- |
| **SARS-CoV-2 infection and natural history parameters** | | |
| Average time between symptom onset and hospitalization, | 3.8 | [1] |
| Mean duration of infectiousness after developing symptoms | 4 | [2, 3] |
| Mean duration of hospitalization | 7 | [4] |
| Mean duration of latent period | 2 | [5, 6] |
| Mean duration of pre-symptomatic period | 1.5 | [7] |
| Relative infectiousness of asymptomatic infected individuals | 1 | [4] |
| Relative infectiousness of hospitalized infected individuals | 0 | Assumed |
| Relative infectiousness of pre-symptomatic infected individuals | 1 | [8] |
| Mean duration of immunity after SARS-CoV-2 first infection | 100 | [9] |
| Mean duration of immunity after SARS-CoV-2 infection for those partially protected ($P$ class) | 180 | [10] |
| Mean duration of immunity for waned vaccinated individuals following a SARS-CoV-2 infection | 100 | [9] |
| Mean duration of vaccine-induced immunity for vaccination with a primary series | 100 | [9] |
| Mean duration of immunity for vaccinated individuals with a primary series following a SARS-CoV-2 infection (hybrid immunity) | 180 | [9] |
| Mean duration of vaccine-induced immunity for vaccination with a booster | 180 | [11,12] |
| Mean duration of immunity for vaccinated individuals with a booster following a SARS-CoV-2 infection (hybrid immunity) | 180 | [11] |
| Proportion of symptomatic infections requiring hospitalization | age-stratified | [13] |
| Relative susceptibility | [0.56, 1, 1, 1, 0.27] | [14] |
| Proportion of symptomatic infections | [0.25, 0.4, 0.4, 0.4, 0.4] | [14] |
| **Protection parameters for partially susceptible individuals** | | |
| Protection against infection for partially susceptible individuals | 0 | [9] |
| Protection against symptomatic infection for partially susceptible individuals | 24.7 | [9] |
| Protection against hospitalization for partially susceptible individuals | 74.6 | [9] |
| **Protection parameters for partially vaccinated individuals** | | |
| Protection against infection for partially susceptible vaccinated individuals | 0 | [9] |
| | 41 | [9] |
| Protection against hospitalization for partially susceptible vaccinated individuals | 95.3 | [9] |

**Table 1.** Description of parameters used in the model.

## References


1. Zhang J, Litvinova M, Wang W, Wang Y, Deng X, Chen X, et al. Evolving epidemiology and transmission dynamics of coronavirus disease 2019 outside Hubei province, China: a descriptive and modelling study. The Lancet Infectious Diseases. 2020;20(7):793–802. doi:10.1016/S1473-3099(20)30230-9.

2. Du Z, Wang L, Xu X, Wu Y, Cowling BJ, Meyers LA. The serial interval of COVID-19 from publicly reported confirmed cases. Emerging Infectious Diseases. 2020;26(6):1341–1343.

3. Bi Q, Lessler J, Eckerle I, Lauer SA, Kaiser L, Vuilleumier N, et al. Insights into household transmission of SARS-CoV-2 from a population-based serological survey. Nat Commun. 2021;12(1):3643. doi:10.1038/s41467-021-23733-5.

4. CDC. COVID-19 Pandemic Planning Scenarios;. Available from: `https://www.cdc.gov/coronavirus/2019-ncov/hcp/planning-scenarios.html`.

5. Lauer SA, Grantz KH, Bi Q, Jones FK, Zheng Q, Meredith HR, et al. The incubation period of coronavirus disease 2019 (COVID-19) from publicly reported confirmed cases: Estimation and application. Annals of Internal Medicine. 2020;doi:10.7326/M20-0504.

6. Zhang J, Litvinova M, Liang Y, Wang Y, Wang W, Zhao S, et al. Changes in contact patterns shape the dynamics of the COVID-19 outbreak in China. Science. 2020;368(6498):1481–1486.

7. Wei WE, Li Z, Chiew CJ, Yong SE, Toh MP, Lee VJ. Presymptomatic Transmission of SARS-CoV-2-Singapore. Morbidity and Mortality Weekly Report. 2020;69(14):411–415.

8. Slifka MK, Gao L. Is presymptomatic spread a major contributor to COVID-19 transmission? Nature Medicine. 2020;26(10):1531–1533. doi:10.1038/s41591-020-1046-6.

9. Bobrovitz N, Ware H, Ma X, Li Z, Hosseini R, Cao C, et al. Protective effectiveness of previous SARS-CoV-2 infection and hybrid immunity against the omicron variant and severe disease: a systematic review and meta-regression. The Lancet Infectious Diseases;doi:10.1016/S1473-3099(22)00801-5.

10. Altarawneh HN, Chemaitelly H, Hasan MR, Ayoub HH, Qassim S, AlMukdad S, et al. Protection against the Omicron Variant from Previous SARS-CoV-2 Infection. New England Journal of Medicine. 2022;386(13):1288–1290. doi:10.1056/NEJMc2200133.

11. Feikin DR, Higdon MM, Abu-Raddad LJ, Andrews N, Araos R, Goldberg Y, et al. Duration of effectiveness of vaccines against SARS-CoV-2 infection and COVID-19 disease: results of a systematic review and meta-regression. The Lancet. 2022;399(10328):924–944. doi:https://doi.org/10.1016/S0140-6736(22)00152-0.

12. Chemaitelly H, Nagelkerke N, Ayoub HH, Coyle P, Tang P, Yassine HM, et al. Duration of immune protection of SARS-CoV-2 natural infection against reinfection. Journal of Travel Medicine. 2022;29(8). doi:10.1093/jtm/taac109.

13. Ferguson NM, Laydon D, Nedjati-Gilani G, Imai N, Ainslie K, Baguelin M, et al. Impact of non-pharmaceutical interventions (NPIs) to reduce COVID-19 mortality and healthcare demand. 2020;.

14. Viner RM, Mytton OT, Bonell C, Melendez-Torres GJ, Ward J, Hudson L, et al. Susceptibility to SARS-CoV-2 infection among children and adolescents compared with adults: A systematic review and meta-analysis. JAMA Pediatrics. 2021;175(2):143–156. doi:10.1001/jamapediatrics.2020.4573.
