# Supplementary material for: Covid19Vaxplorer: A free, online, user-friendly COVID-19 vaccine allocation comparison tool
Source: PLOS Glob Public Health. 2024 Jan 22;4(1):e0002136. doi: 10.1371/journal.pgph.0002136 (PMC10802966; doi:10.1371/journal.pgph.0002136)
Supplement: S2 Table — (PDF) [file pgph.0002136.s011.pdf]

Vaccine effectiveness						
	Omicron (Primary series)			Omicron (booster)		
Vaccine	Hospitalization VE_{H}	Infection VE_{SUS}	Symptomatic infection VE_{SYMP}	Hospitalization VE_{H}	Infection VE_{SUS}	Symptomatic infection VE_{SYMP}
AstraZeneca	0.71	0.36	0.29	0.94	0.63	0.63
Cansino	0.48	0.32	0.26	0.66	0.62	0.66
CoronaVac	0.37	0.24	0.26	0.5	0.47	0.65
Covaxin	0.57	0.38	0.31	0.78	0.73	0.78
Jansen	0.57	0.33	0.26	0.86	0.72	0.67
Moderna	0.73	0.48	0.36	0.97	0.92	0.92
Novavax	0.65	0.43	0.36	0.89	0.83	0.90
Pfizer/BioNTech	0.72	0.44	0.30	0.95	0.86	0.88
Vero Cell	0.53	0.35	0.31	0.73	0.68	0.78
Sputnik-V	0.67	0.44	0.36	0.92	0.86	0.92

Table 1. Default vaccine effectiveness values used in **Covid19Vaxplorer**, taken from [1]

References

1. Institute for Health Metrics Evaluation. COVID-19 vaccine efficacy summary; 2022. `https://www.healthdata.org/covid/covid-19-vaccine-efficacy-summary`.
