# Supplementary material for: Covid19Vaxplorer: A free, online, user-friendly COVID-19 vaccine allocation comparison tool
Source: PLOS Glob Public Health. 2024 Jan 22;4(1):e0002136. doi: 10.1371/journal.pgph.0002136 (PMC10802966; doi:10.1371/journal.pgph.0002136)
Supplement: S3 Table — (PDF) [file pgph.0002136.s012.pdf]

**Vaccine**	**Group 1 0-19**	**Group 2 20-49**	**Group 3 50-64**	**Group 4 65-74**	**Group 5 75+**
Covishield	0	0	0	0	250,000
Unknown	0	0	0	56,154	23,929
Sinopharm	0	0	648,845	701,155	0
AstraZeneca	0	0	210,000	0	0
Covaxin	0	0	250,000	0	0
Jansen	0	7,270,765	1,343,991	0	0
**Total**	**0**	**7,270,765.000**	**2,452,836.000**	**757,309.000**	**273,929.000**

Table 1. Assumed distribution of COVID-19 prior vaccination in Afghanistan per age group and vaccine product.
