# Supplementary material for: Covid19Vaxplorer: A free, online, user-friendly COVID-19 vaccine allocation comparison tool
Source: PLOS Glob Public Health. 2024 Jan 22;4(1):e0002136. doi: 10.1371/journal.pgph.0002136 (PMC10802966; doi:10.1371/journal.pgph.0002136)
Supplement: Attachment — Submitted filename: PGPH-D-23-01112_reviewer.pdf [file pgph.0002136.s013.pdf]

PLOS Global Public Health

Covid19Vaxplorer: a free, online, user-friendly COVID-19 Vaccine Allocation Comparison Tool

--Manuscript Draft--

**Manuscript Number:**	PGPH-D-23-01112
**Article Type:**	Research Article
**Full Title:**	Covid19Vaxplorer: a free, online, user-friendly COVID-19 Vaccine Allocation Comparison Tool
**Short Title:**	Covid19Vaxplorer a free, online, user-friendly COVID-19 Vaccine Allocation Comparison Tool
**Corresponding Author:**	Laura Matrajt<br>Fred Hutchinson Cancer Research Center<br>Seattle, UNITED STATES
**Order of Authors:**	Imelda Trejo
	Pei-Yao Hung
	Laura Matrajt
**Keywords:**	COVID-19, vaccine, vaccine allocation, mathematical model, online tool
**Abstract:**	Background<br><br>There are many COVID-19 vaccines currently available, however, Lowand middle-income countries (LMIC) still have large proportions of their populations unvaccinated. Decision-makers need to take decisions as of how best to allocate available vaccine[illegible] (e.g. boosters or primary series vaccination, which age groups to target) but LMIC often lack the resources to undergo quantitative analyses of vaccine allocation, resulting and ad-hoc policies. We developed Covid19Vaxplorer (https://covid19vaxplorer.fredhutch.org/), a free, user-friendly online tool that simulates region-specific COVID-19 epidemics in conjunction with vaccination.<br><br>Methods<br><br>We developed an age-structured mathematical model of SARS-CoV-2 transmission and COVID-19 vaccination. The model considers vaccination with up to three different vaccine products, primary series and boosters. We simulate partial immunity derived from waning of natural infection and vaccination. The model is embedded in an an online tool that allows users in 183 regions in the world to compare several vaccination strategies simultaneously, adjusting parameters to their local epidemics, infrastructure and logistics, and reports cumulative number of deaths and epidemic curves.<br><br>Results<br><br>We provide two usage examples of Covid19Vaxplorer for vaccine allocation in Haiti and Afghanistan, which had as of Spring 2023 2% and 33% of their populations vaccinated, and show that in these examples, giving primary series vaccinations prevents more deaths than boosters. Covid19Vaxplorer is an online, free, user-friendly tool that facilitates evidence-based decision making for vaccine distribution.
**Additional Information:**	
**Question**	**Response**
**Financial Disclosure**<br><br><br><br>Enter a financial disclosure statement that describes the sources of funding for the	This work was supported by the National Science Foundation under Grant No.2210382. This work was partially supported by grants from the National Institutes of Health (UM1AI068635 and UM1AI068617). L.M. was also supported by a grant from Centers for Disease Control and Prevention (NU38OT000297-02) through their cooperative agreement with the Council of State and Territorial Epidemiologists. The funders The funders had no role in study design, analysis, decision to publish, or

Powered by Editorial Manager® and ProduXion Manager® from Aries Systems Corporation

work included in this submission and the role the funder(s) played. This includes grants and any commercial funding of the work or authors.<br><br>This statement will be typeset if the manuscript is accepted for publication.<br><br>*Please review the submission guidelines and the instructions link below for detailed requirements and guidance.*	preparation of the manuscript.
**Competing Interests**<br><br>On behalf of all authors, disclose any competing interests that could be perceived to bias this work.<br><br>This statement will be typeset if the manuscript is accepted for publication.<br><br>*Please review the instructions link below and PLOS Global Public Health's competing interests policy to determine what information must be disclosed at submission.*	We declare no competing interests.
**Data Availability**<br><br>Before publication, Authors are required to make fully available and without restriction all data underlying their findings. Please see our PLOS Data Policy page for detailed information on this policy.<br><br>A **Data Availability Statement**, detailing where the data can be accessed, is required at first submission. Insert your Data Availability Statement in the box below.<br><br>Please see the data reporting section of our submission guidelines for instructions	All the Data used in this work is publicly available and given in the references. The code will be available at: https://github.com/FredHutch/covid19vaccinesimulator

on what you need to include in your Data Availability Statement.<br><br>This statement will be typeset if the manuscript is accepted for publication.<br><br>*PLOS allows rare exemptions to address legal and ethical concerns. If you have legal or ethical restrictions, please use the box below to detail these in full sentences for the Journal team to consider.*	

Powered by Editorial Manager® and ProduXion Manager® from Aries Systems Corporation

Manuscript
Click here to access/download;Manuscript;main.pdf

Covid19Vaxplorer: a free, online, user-friendly COVID-19 Vaccine Allocation Comparison Tool

Imelda Trejo[1,†], Pei-Yao Hung[2], Laura Matrajt[1,3,*,†]

June 9, 2023

[1] Vaccine and Infectious Disease Division, Fred Hutchinson Cancer Center, Seattle, WA, US
[2] Institute For Social Research, University of Michigan, Ann Arbor, MI, US
[3] Department of Applied Mathematics, University of Washington, Seattle, WA, US
[*] Corresponding Author
[†] These authors equally contributed to this work.

Abstract

Background: There are many COVID-19 vaccines currently available, however, Low- and middle-income countries (LMIC) still have large proportions of their populations unvaccinated. Decision-makers need to take decisions as of how best to allocate available vaccine (e.g. boosters or primary series vaccination, which age groups to target) but LMIC often lack the resources to undergo quantitative analyses of vaccine allocation, resulting and ad-hoc policies. We developed **Covid19Vaxplorer** (https://covid19vaxplorer.fredhutch.org/), a free, user-friendly online tool that simulates region-specific COVID-19 epidemics in conjunction with vaccination.

Methods: We developed an age-structured mathematical model of SARS-CoV-2 transmission and COVID-19 vaccination. The model considers vaccination with up to three different vaccine products, primary series and boosters. We simulate partial immunity derived from waning of natural infection and vaccination. The model is embedded in an an online tool that allows users in 183 regions in the world to compare several vaccination strategies simultaneously, adjusting parameters to their local epidemics, infrastructure and logistics, and reports cumulative number of deaths and epidemic curves.

Results: We provide two usage examples of **Covid19Vaxplorer** for vaccine allocation in Haiti and Afghanistan, which had as of Spring 2023 2% and 33% of their populations vaccinated, and show that in these examples, giving primary series vaccinations prevents more deaths than boosters. **Covid19Vaxplorer** is an online, free, user-friendly tool that facilitates evidence-based decision making for vaccine distribution.

Introduction

The COVID-19 pandemic has killed more than 6.8 million people worldwide as of March 2023 [1]. There are more than 20 vaccines currently available with various levels of vaccine effectiveness [2]. High-income countries (HIC) have vaccinated and boosted several times the majority of their eligible populations, but only 28% of the population of low income countries have received at least one dose of vaccine. As of March 2023, some low-income countries (LIC) (e.g. Haiti, Senegal) have less than 10% of their population fully vaccinated [3]. COVAX, a global effort co-led by the Coalition for Epidemic Preparedness Innovations (CEPI), the Global Vaccine Alliance (Gavi) and the World Health Organization (WHO), was established to distribute donated vaccines to low- and middle-income countries. Because on their dependence on COVAX, LMIC have received, and are likely to continue to receive, a portfolio of several vaccine products, with very different efficacies against specific variants, forcing public health officials in these regions to take decisions about how to best allocate these resources. However, many of these countries lack the resources to develop quantitative models to evaluate possible vaccine allocations, resulting in an ad-hoc allocation of precious resources. There has been an extensive number of mathematical models published for COVID-19, and some online tools were developed for simulating SARS-CoV-2 transmission [4–6]. However, to our knowledge, these tools are usually centered in a single country, state or region. Moreover, most of the research has been centered around HIC, with fewer examples considering LMIC. Existing online tools either have fixed worldwide epidemic projections (number of deaths, hospital resource use, infections) that are not interactive or they have very limited capabilities for user-input, notably not allowing the user to select multiple vaccines with different efficacies. More importantly, as the pandemic has entered an endemic phase in HIC, and the urgency for modeling vaccine allocation has dissipated in these countries, most of these tools are no longer maintained.

We developed **Covid19Vaxplorer** (https://covid19vaxplorer.fredhutch.org/), a free, user-friendly tool that simulates region-specific COVID-19 epidemics in conjunction with vaccination campaigns with multiple vaccine products. The aim of our tool is to provide decision-makers in different regions in the world a general framework for vaccine allocation guidance and comparison, allowing public health officials in 183 regions (representing $\geq$97.2% of the global population) to simulate regional epidemics based on local demographics, epidemiology, logistical constraints, and vaccine availability. The tool produces key outcomes including expected numbers of deaths, hospitalizations and cases, with the possibility of simulating several scenarios of vaccine allocation at once for a side-by-side comparison. The tool is highly flexible and customizable, while remaining very user-friendly.

Methods

Model

Basic model structure

Based on [7, 8], we developed an age-structured deterministic mathematical model of SARS-CoV-2 transmission and vaccination with up to three vaccine products (referred as "vaccines" for simplicity below). The model accounts for infections, reinfections, waning naturally- and vaccine acquired immunity, primary vaccination series and boosters (single boosters). We divide the population into five age groups: 0–19, 20–49, 50–64, 65–74, and those 75 and older. Each age-group is partitioned into nine compartments depending on their vaccination status and degree of protection: unvaccinated (indexed by $\alpha = i$), partially protected with naturally-induced immunity (indexed by $\alpha = P_i$), or with vaccine-acquired waned immunity, (indexed by $\alpha = W_i$), vaccinated with primary series with vaccine product j (indexed by $\alpha = V_{ij}$), and vaccinated and boosted with vaccine product j (indexed by $\alpha = B_{ij}$), $i = 1, 2, \ldots, 5$, and $j = 1, 2, 3$ (we do not distinguish between different vaccines for primary doses in boosted individuals and assume that only the booster defines the protection of the individual). Each of previous compartments is further stratified depending on their disease status: susceptible (S_α), exposed (E_α) (infected but not yet infectious), asymptomatic infected (A_α), pre-symptomatic infected (P_α), symptomatic infected (I_α), symptomatic hospitalized (H_α), and removed (asymptomatic, symptomatic or hospitalized). The removed compartments were split into two phases to allow for a slower representation of the loss of immunity: removed asymptomatic (RA_α or RRA_α), removed symptomatic (R_α or RR_α) and removed hospitalized (RH_α or RRH_α). A proportion of those in the removed hospitalized compartment RH_α die and are removed from the population. In our model, individuals contact each other in four possible locations: home, work, school and community. We use the values given in [9] to simulate these contacts. This gives rise to a system with 540 homogeneous compartments. A diagram of the model is given in Fig 1. The equations for the model and default parameters can be found in the Supplemental Material.

Modeling vaccine effectiveness

We assumed leaky vaccines [10], that have three potential effects: a reduction in the susceptibility to infection VE_{SUS}, a reduction in the probability of developing symptoms given infection VE_{SYMP} and a reduction in the probability of developing severe symptoms given symptomatic infection VE_{SEV}. In addition, we assume that those with waned immunity (either naturally-acquired or vaccine-induced immunity) would retain some protection against infection, symptomatic infection and/or severe infection.

Modeling vaccination campaigns

We model vaccination campaigns with a primary series or boosters. We do not model individual doses for primary series. We assume that infected symptomatic individuals will not get vaccinated, so that, at the beginning of each vaccination campaign, we distribute available vaccine among all eligible compartments (susceptible S_α, exposed E_α, asymptomatic infected A_α, pre-symptomatic infected P_α, and all the recovered compartments: RA_α, RRA_α, R_α, RR_α, RH_α, RRH_α, where $\alpha = i, P_i, W_i, V_{ij}$ or B_j with $i = 1, 2, \ldots, 5$ and $j = 1, 2, 3$) proportionally to their relative size at that point in time. Vaccinated individuals from compartments E_α, A_α, and P_α do no acquire any type of vaccine protection. We calculate the duration of the vaccination campaign according to the daily vaccination rate provided by the user.

In our model, all individuals who have been previously vaccinated are moved to the boosted corresponding classes when boosted. That is, irrespective of their current immunity state (waned or not) we assume that a booster will renew their protection. For example, individuals in compartments $S_{V_{ij}}$ and S_{P_i} boosted with vaccine product k would go to the booster compartment B_{ik}. We assume that a single infection confers similar immunity as a primary series vaccination, so that when individuals in the recovered classes ($RA_i, RRA_i, R_i, RR_i, RH_i$ and RRH_i, with $i = 1, 2, \ldots, 5$) get vaccinated with vaccine product j, they are moved to the boosted compartment $S_{B_{ij}}$. Similarly, individuals who are unvaccinated and have had a single infection for whom immunity has waned (those in the S_{Pi} compartments) are transferred to the corresponding susceptible vaccinated with a primary series of product j, $S_{V_{ij}}$ Those individuals who have one infection, have waned immunity, and get vaccinated with vaccine product j after a second infection (those in the $RA_{Pi}, RRA_{Pi}, R_{Pi}, RR_{Pi}, RH_{Pi}, RRH_{Pi}$ compartments) are moved to the vaccinated boosted compartment $S_{B_{ij}}$ with $i = 1, 2, \ldots, 5, j = 1, 2, 3$ and $k = 1, 2, 3$.

Figure S1: Flow diagram of the disease progression and vaccine allocations across susceptible (S_α), exposed (E_α), asymptomatic (A_α), pre-symptomatic (P_α), symptomatic (I_α) hospitalized (H_α), removed symptomatic ($R\alpha$, $RR\alpha$), removed asymptomatic ($RA\alpha$, $RRA\alpha$), removed hospitalized (RH_α, RRH_α) compartments, $\alpha = i, P_i, W_i, V_{ij},, V_{ij}$. A proportion of those in the hospitalized removed compartments are assumed to die and are removed from the simulation (arrows omitted for clarity).

Design Process

Covid19Vaxplorer was developed through a user-centered design (UCD) [11] process with the goal of creating a user-friendly tool for supporting non-technical users, including health officials, to design vaccine allocation strategies that could lead to a better outcome. It was implemented using modern web frameworks, Vue.js [12] and Flask [13]. With the goal of supporting non-technical users such as health officials with this tool, the team, which consists of both human-centered researcher and mathematical modelers who have experience working with health officials, met regularly to gather requirements on the intended utility of this tool and

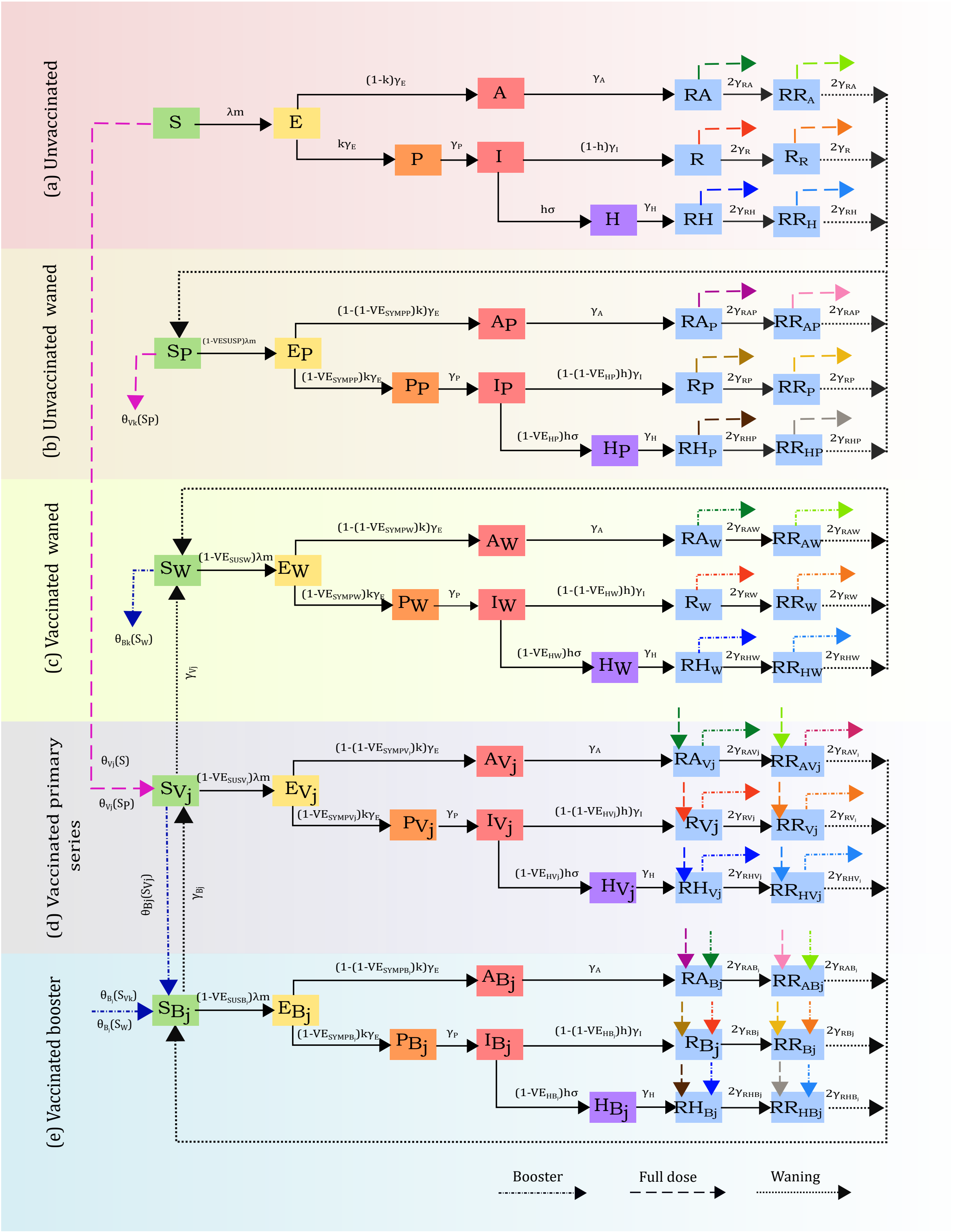

Figure 1: Flow diagram of the disease progression and vaccine allocation.

the rationale for having different parameters needed by the model.

Because a large number of parameters are required (191 parameters per strategy), as is usually the case with mathematical models of infectious disease transmission (e.g. [14, 15], we apply the concept of information architecture [16], which emphasizes the importance of organizing information (e.g., parameters in our case) and providing navigation facility in a way that aligns with users' mental model and workflow.

Our team adapted the card sorting technique [16] as a way to build consensus as to how users might conceptually categorize all the parameters needed by the model and to provide a logical flow for specifying different categories of parameters. The team consolidated the proposed information architecture using paper prototyping [17] and created a low-fidelity prototype (i.e., paper-and-pencil sketches) as an artifact to iterate on the design.

Covid19Vaxplorer was designed to avoid saturation and screen tiredness in its users. To achieve this, **Covid19Vaxplorer** helps them navigate different sets of parameters, starting with basic and simple population characteristics, to a more complicated set of parameter definitions that are required to characterize the vaccine availability and prioritization scheme. The proposed interaction flow consists of 4 sections: "Location", "Vaccine", "Vaccine Planning", and "Outcome".

First, the workflow starts with known information about the region of interest, represented as a small set of parameters, including population, the level of social distancing, and current/past infection statistics.

Second, **Covid19Vaxplorer** prompts users to enter vaccine-related information, which involves a larger number of parameters compared to the first phase, such as the types of vaccines that have been used to date and that will be available for future use, their effectiveness, and the number of people in each age group who were previously vaccinated.

Third, users are invited to specify planning-related parameters, including vaccine availability (what and when), and how vaccines will be allocated to different age groups within the population. Compared to the second "vaccine" phase, here users are expected to actively operationalize their vaccine allocation approaches into the parameters provided by this tool, before seeing the results.

Finally, users are presented with projections of key epidemic outcomes based on the information they input. They can explore variations of vaccine allocation strategies to compare the outcomes and evaluate which strategy is more effective. Users are able to go back to any phase to update parameters so that they can understand how those changes affect the projections associated with each allocation strategy.

In the next section we describe in more detail each of the phases.

Tool Usage

Covid19Vaxplorer allows users to input region-specific parameters (types of vaccines, number of doses, vaccination rate, circulating variants, demographics, non-pharmaceutical interventions) and to compare several scenarios of vaccine allocation. Below we describe the tool

functionality in full detail.

The tool has 11 main windows divided in four main sections, where each window asks the user to provide specific information:

1. **Location:** In this section, the user provides basic parameters for the region of interest. The section is divided in three windows:

   (a) **Region:** User selects the region they want to simulate among 183 regions, and the basic reproduction number R_0 (defined as the average number of secondary infections an infected individual will generate in a fully susceptible population). Suggested values of R_0 are provided for several SARS-CoV-2 variants, based on the numbers given on [18].

   (b) **Social distancing:** User selects how people are practicing social distancing at each location (home, work, school, community) if people are practicing social distancing, ranging from "fully social distanced" to "not at all" social distanced. These inputs are translated to the model as multipliers to each of these contact matrices.

   (c) **Infection Prevalence:** In this window, the user inputs the proportion of people in each age group who have been previously infected (and who then will have some protection against reinfection, symptoms and/or severe disease) and those who are currently infected. The window provides default percentages for cumulative prior infection as of November 2021 taken from [19].

2. **Vaccine:** The vaccine section of the tool is divided in four separate windows to facilitate the users' input of basic vaccine-related parameters.

   (a) **Options:** Here, the user selects up to three vaccine products that will be used in the simulation. The user can choose among 10 pre-loaded vaccine products (the most common vaccine products available in the market for which vaccine effectiveness is readily available) or can create their own vaccine product (Figure 2).

   (b) **Previous Vaccination:** In this window, users input for each age group and each vaccine product, the percentage of that group that has been previously vaccinated either with a primary series or a booster.

   (c) **Vaccine effectiveness:** Here, the user inputs the vaccine effectiveness for each vaccine product that was previously selected. For convenience, default values are given for the majority of pre-loaded vaccine products, taken from [20].

3. **Vaccine Planning:** This section is divided in three windows that facilitate the input of data and parameters by the user:

   (a) **Availability:** The user inputs the amount of up to three vaccine products that will be available (same products used in the "Previously vaccinated" window above), the date when they will be available and the number of vaccines that will be administered per day (vaccination rate).

(b) **Allocation strategy:** For each vaccine product, the user is presented with two tables to allocate the available vaccine courses: the primary series table and the booster table. For each table, the user indicates the percentage of vaccine courses to be given as primary series and/or boosters. Then, the user further determines the percentage of vaccine to be given to unvaccinated individuals in each age group as primary series (Primary Series table), and the percentage of vaccine to be given in each age group to previously vaccinated individuals as boosters (Boosters table). **Covid19Vaxplorer** includes features like the toolbox button (that resets either the Primary series table or the Booster table for any given vaccine) allowing users to quickly and easily input their vaccine allocation strategy (Figure 3).

(c) **Period for simulation:** The user provides the beginning and end dates for the simulation. Default end dates are provided based on the amounts of vaccine available and vaccination rates, but the simulation end date can be extended beyond that date.

(d) **Additional parameters:** This window presents a table with the additional parameters (e.g. duration of infectious period, duration of naturally-acquired immunity, proportion of asymptomatic infections) needed to run the simulation. Default parameter are provided (values given in Table S1).

4. **Outcome:** This is where the results are displayed and visualized. The following key outcomes are given: Cumulative number of deaths over the simulation period, Maximum number of hospitalized individuals, epidemic curves for the daily number of deaths, hospitalizations, symptomatic infections and infections.

Covid19Vaxplorer allows the user to Edit Duplicate, or Delete a given vaccination strategy, and displays the outcomes in plots next to the original strategy, so that comparisons are easily handled. In addition, the user can use the Export button to download the output of the simulation for further analysis.

Note that while **Covid19Vaxplorer** provides default parameters for all the parameters needed to run the model (natural history parameters, vaccine-induced and naturally acquired immunity parameters, cumulative infections, etc), all of these parameters are customizable by the user, so that they can adapt the tool to their particular needs and circumstances.

Results

In this section, we provide two examples of the usage of **Covid19Vaxplorer** and how this can help decision-makers. We consider two possible strategies: **Strategy 1**: to give as many boosters as possible (referred below as **"Boosters first"** and **Strategy 2**: To give as many primary series as possible (referred below as **"Primary series first"**). For both strategies, vaccination starts with older age groups and goes in decreasing order by age. For example, under in Strategy 1, we would start vaccinating the oldest age group (group 5, 75 years old and older), followed

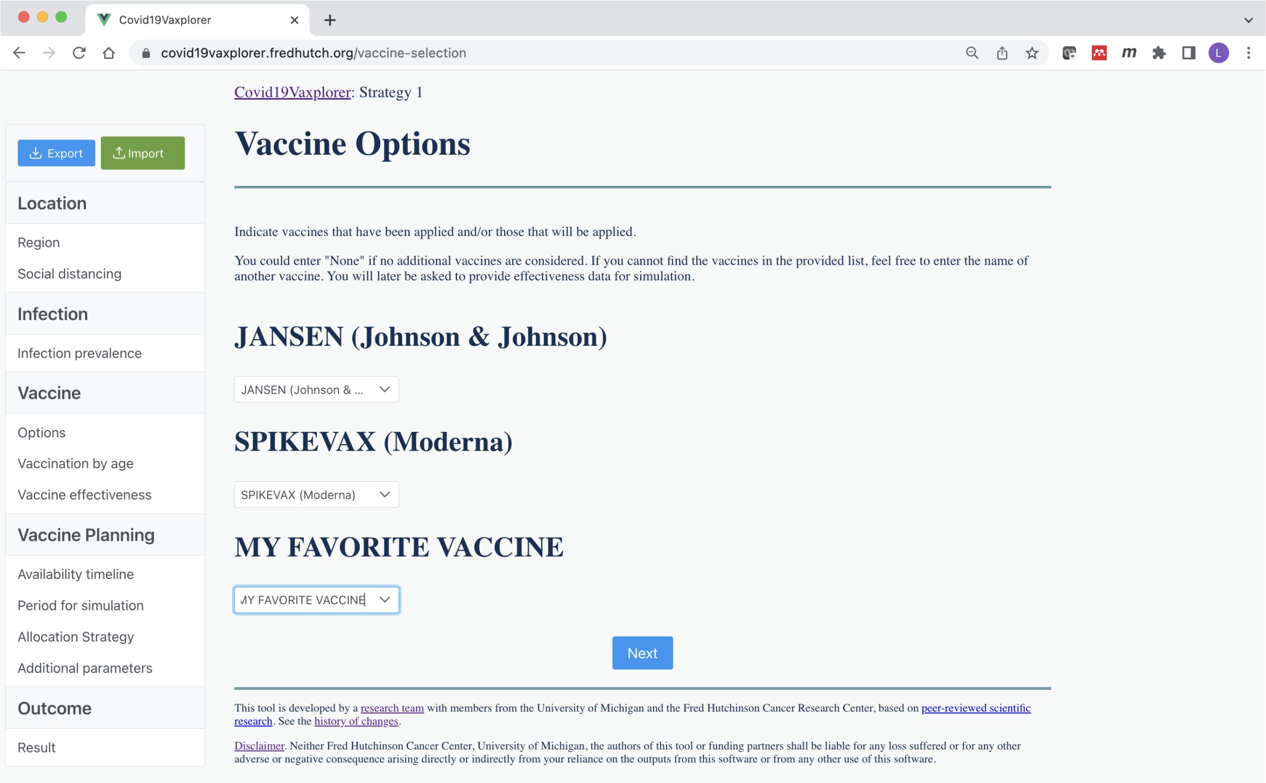

Figure 2: Example of the Vaccine Options window in **Covid19Vaxplorer**. The user can select up to three vaccine products for their simulation, to choose among 10 pre-loaded vaccines or they can input their own vaccine. For each vaccine product, there is a corresponding vaccine effectiveness table.

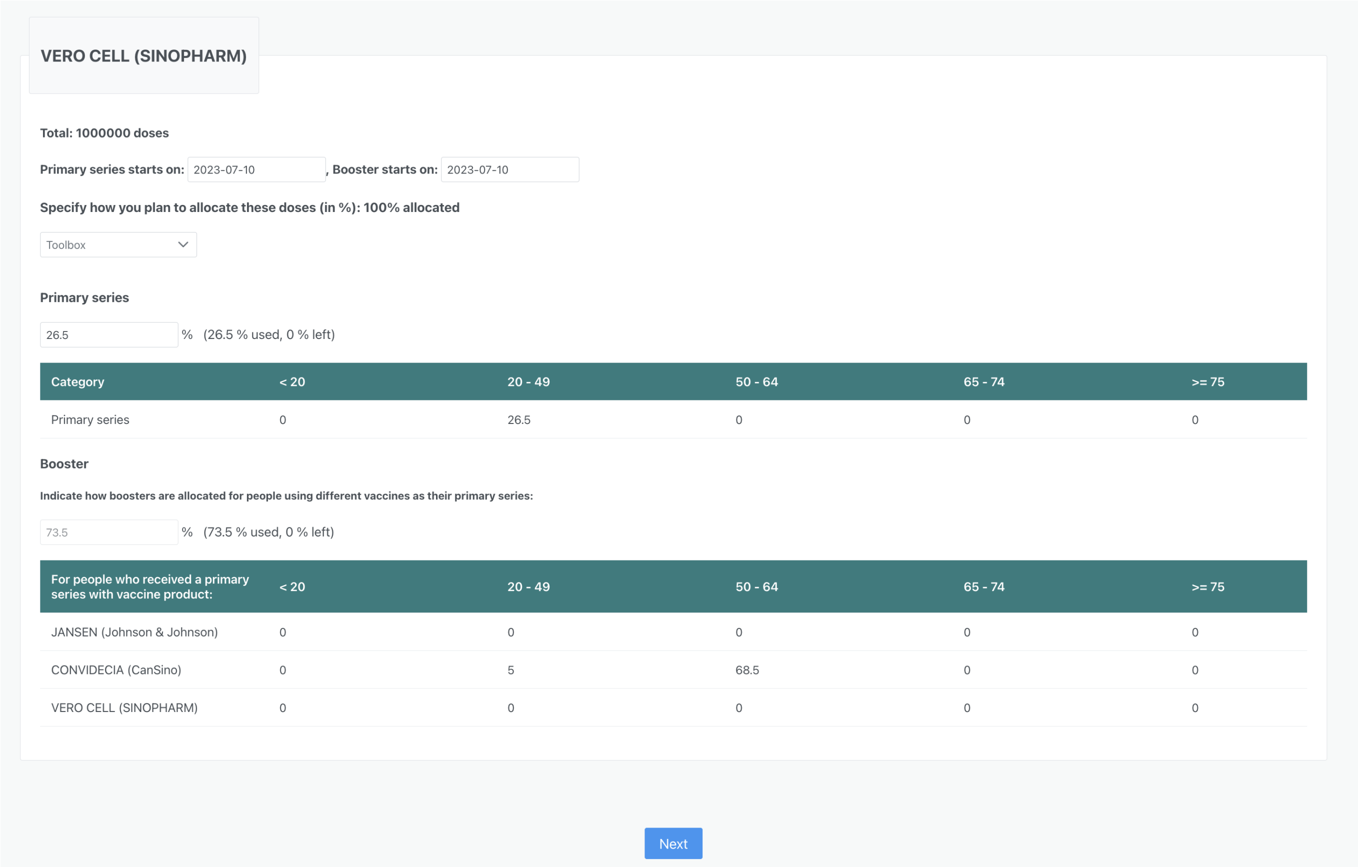

Figure 3: Example of the vaccine allocation window in **Covid19Vaxplorer**. For each vaccine given, the user is presented with two tables to allocate the available vaccine. The user indicates the percentage of available vaccine to be given as primary series and the percentage to be given as boosters. Within each table, the user then determines what percentage of vaccine will be allocated as primary series for each age group, and what percentage of vaccine will be allocated as boosters among age groups and previously vaccinated groups.

by those in group 4 (65-75 years old), then adults in group 3 (50-65 years old) and so on as vaccine availability permits. For the examples below, we consider a 6 month simulation period.

Hypothetical scenario: Haiti

We explore here a hypothetical scenario for vaccine allocation in Haiti. Based on [19], we assumed that 29.6% of the population in Haiti had pre-existing immunity. While this number is presumably too low, sensitivity analysis with 80% pre-existing immunity showed similar qualitative results with a strategy allocating primary series first outperforming the one prioritizing boosters Figure S3). As of March 2023, 2.06% of the population in Haiti has been fully vaccinated [21]. Since we could not find any specific information about which age groups had been previously vaccinated or about which vaccine products have been previously used, we assumed the following:

- Health-care professionals were prioritized to be vaccinated first. There are approximately 16,000 health care professionals in Haiti [22], and we assumed them to be distributed among the adult age groups proportionally to the population in each of those age groups.
- We assume that the older age groups (75 and older and 65-75 years old) were also prioritized to receive vaccination first as availability permitted after health care workers were vaccinated, starting with the oldest age group first.
- Based on news reports, we assume that the first vaccine product given in Haiti was Spikevax (Moderna).

We consider a hypothetical situation in which Haiti would experience a new SARS-CoV-2 wave with a variant with similar infectivity as the delta variant ($R_0 = 3$) with 0.1% of the population currently infected. Furthermore, this region would receive an additional 3.5 Million vaccine courses over the following 12 weeks split as follows:

- 1 Million courses of SPIKEVAX vaccine available immediately,
- 1.5 Million courses of CONVIDECIA (Cansino) vaccine available 7 weeks after receiving the SPIKEVAX vaccines,
- 1 Million courses of VERO CELL (Sinopharm) vaccine 4 weeks after receiving the CONVIDECIA vaccine.

We assume that for all three vaccines, 50,000 individuals can be vaccinated daily. Figure S1 shows the percentage of each age group that would be vaccinated with primary series or boosters under each strategy considered.

Using **Covid19Vaxplorer**'s capability to compare strategies back to back, we can determine the best vaccination strategy. We simulated both strategies over a 6 month period. Figure 4

shows the output of **Covid19Vaxplorer** comparing these two strategies. Under the **Boosters first** strategy (Strategy 1) there would be 18,223 cumulative deaths at the end of the simulation period and there would be 6,852 hospitalizations at the peak of the epidemic wave. In contrast, under the **Primary series first** strategy (Strategy 2), there would be 14,553 cumulative deaths at the end of the simulation period and 6,856 hospitalizations at peak. Hence, while the maximum number of hospitalizations is similar, there would be over 3,500 more deaths if a strategy favoring boosters was implemented.

Hypothetical scenario: Afghanistan

Our second example shows a hypothetical scenario for vaccination in Afghanistan. Based on [19], we assumed that 84.8% of the population was previously infected. As of March 2023, 33% of the population had been fully vaccinated in Afghanistan [21]. Based on the UNICEF COVID-19 Market Dashboard [23], 19,724,217 vaccine doses have been assigned to Afghanistan as of February 2023, with a combination of vaccine products: Sinopharm, Vaxzevira, Covaxin, Jansen and an unknown vaccine product [23]. Based on [21], we assumed that 10,754,839 of those have been administered, all of them as primary series. We assumed that vaccine products were administered in the order they were received, and that the oldest age groups were vaccinated first, and younger age groups were vaccinated in decreasing order. Table S3 shows the distribution of previously vaccinated individuals among age groups and vaccine products. For this example, we assume that the difference between the vaccine doses that have been assigned and those that have been administered (6,630,189 doses, all Jansen vaccine) would be administered over the next 6 months, and we compared, as described above, two vaccination strategies: the**"Boosters first" (Strategy 1)** and **"Primary series first" (Strategy 2)** strategies. Because **Covid19Vaxplorer** allows three different vaccine products at a time, we combined vaccine products with similar vaccine effectiveness, so that we formed three groups: Vaccine 1 with Vaxzevira, Vaccine 2 with Sinopharm, Covaxin and Unknown, and Vaccine 3 with Jansen. Figure S2 shows the two hypothetical distributions by age and previously vaccinated groups.

Figure 5 shows a screenshot from the output page of **Covid19Vaxplorer**. In this example, vaccinating as many people with a primary series is still better than using the vaccine in boosters, with 823 less deaths in the former than the latter (23,084 vs 23,907 respectively). Moreover, the **"Primary series first"** strategy would result in a much lower hospitalization peak: 5,647 hospitalizations at peak compared to 8,519 for the **"Boosters first"** strategy. This could be important because it might allow public health officials to maintain the healthcare system within working capacity.

Discussion

The COVID-19 pandemic has highlighted the need to use quantitative tools to take evidence-based decisions for vaccine distribution. As the pandemic has evolved into an endemic period,

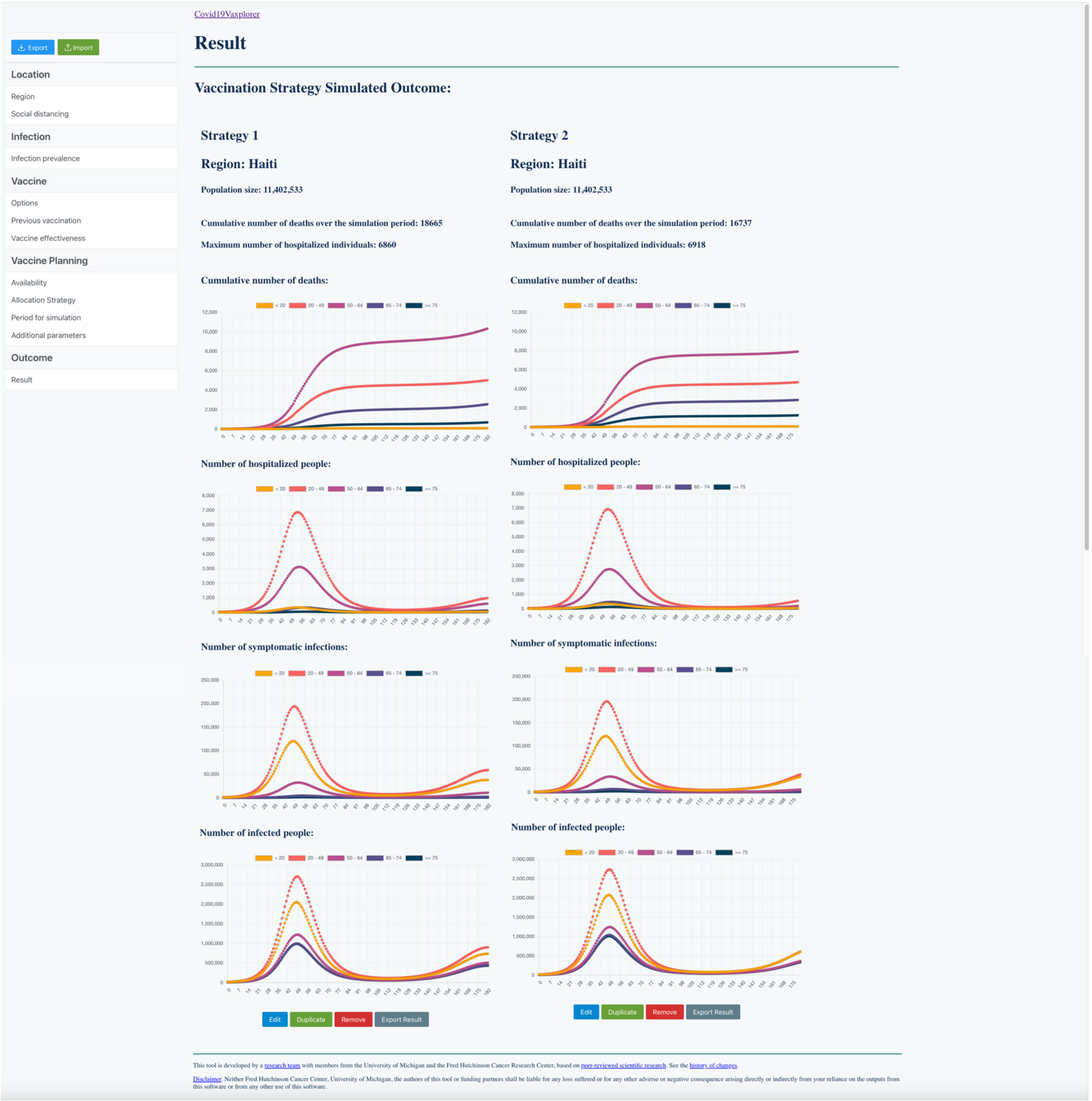

Figure 4: **Covid19Vaxplorer** results page for comparing two strategies in a hypothetical situation in Haiti. Strategy 1, "boosters first" strategy, would result in 18,665 cumulative deaths at the end of the simulation period and 6860 hospitalizations at the peak. In contrast, Strategy 2, "primary doses first" would result in 16,737 cumulative deaths and 6,918 hospitalizations at the peak over the same simulation period.

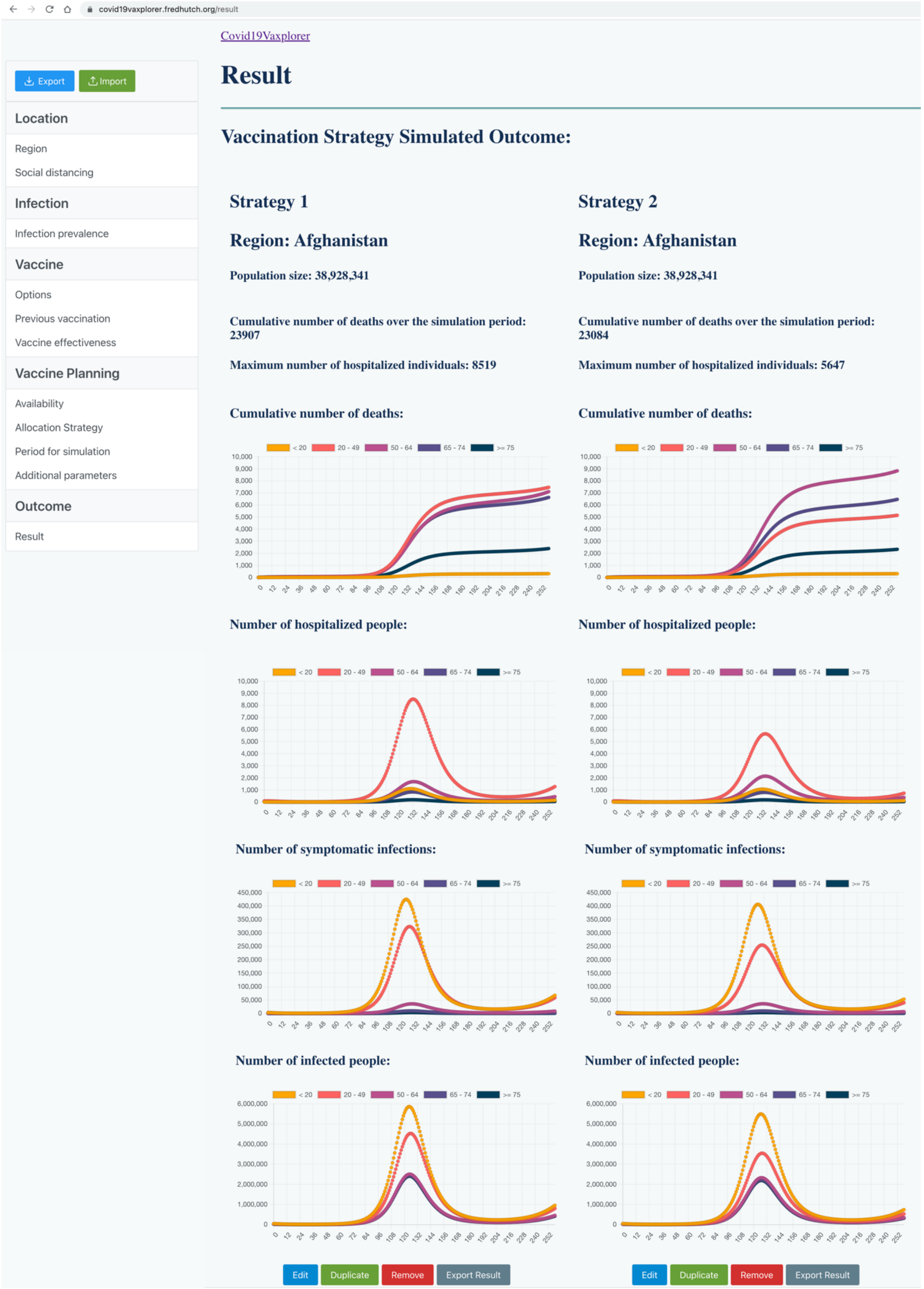

Figure 5: **Covid19Vaxplorer** results page for comparing two strategies in a hypothetical situation in Afghanistan. Strategy 1, “boosters first” strategy, would result in 23907 cumulative deaths at the end of the simulation period and 8519 hospitalizations at the peak of the epidemic wave. In contrast, Strategy 2, “primary doses first” would result in 23084 cumulative deaths and 5647 hospitalizations at the peak.

important questions remain regarding COVID-19 vaccine allocation. In particular, decision-makers in LMIC, where large proportions of the population remain unvaccinated and or un-boosted, might face difficult decisions regarding how to allocate COVID-19 vaccine doses: to give boosters to their high-risk populations or to vaccinate with a primary series their unvaccinated populations. Yet, many countries lack the resources to quantitatively compare different vaccination strategies. **Covid19Vaxplorer**, an online tool to simulate and compare different COVID-19 vaccination strategies, was developed to fill this gap, so that decision-makers around the world have access to a simple tool allowing them to make informed, evidence-based decisions.

We developed **Covid19Vaxplorer** with three main factors in mind. First, **Covid19Vaxplorer** needed to be free, so that anyone in the world could use it. Second, **Covid19Vaxplorer** needed to be online, so that it would be accessible for anyone with internet access. Finally, **Covid19Vaxplorer** needed to be easy to use, so that decision makers, with or without mathematical experience, could use it to compare potential vaccination strategies. This last point proved to be the most difficult to achieve, as we needed to strike a balance between a simple enough tool to be user-friendly and a realistic enough mathematical model to provide useful vaccine allocation comparisons.

Covid19Vaxplorer, as all mathematical models, is subject to several limitations including the following: First, the model does not distinguish between first and second doses and encompass both under a primary series label. While it incorporates product-specific information about the length of vaccine-induced immunity and its effectiveness, it assumes a single duration of immunity for waned vaccinated individuals following a SARS-CoV-2 infection. **Covid19Vaxplorer** is not fitted to any epidemic in particular, rather, it provides generally accepted values for the most common parameters. While the user can modify the parameters for their own region, it is not suited to make projections and it should not be used or construed in such a way. Rather, the value of **Covid19Vaxplorer** relies in its ability to allow users to make back-to-back comparisons of vaccination strategies.

Acknowledgments

We thank Dr. Dobromir Dimitrov for helpful comments while developing this project. This work was supported by the National Science Foundation under Grant No.2210382. This work was partially supported by grants from the National Institutes of Health (grant No. UM1AI068635). L.M. was also supported by a grant from Centers for Disease Control and Prevention (grant No. NU38OT000297-02) through their cooperative agreement with the Council of State and Territorial Epidemiologists.

References

[1] WHO. WHO Coronavirus (COVID-19) Dashboard;. https://covid19.

who.int/?adgroupsurvey={adgroupsurvey}&gclid=CjwKCAjw_
YShBhAiEiwAMomsEBUzmW8Ff5M31-cjdvgGmDS5QxkY-dxZK8LdtWkvtLPMEbyH730EjhoC[illegible]
BwE.

[2] Higdon MM, Wahl B, Jones CB, Rosen JG, Truelove SA, Baidya A, et al. A systematic review of COVID-19 vaccine efficacy and effectiveness against SARS-CoV-2 infection and disease. medRxiv. 2021;doi:10.1101/2021.09.17.21263549.

[3] Mathieu E, Ritchie H, Ortiz-Ospina E, Roser M, Hasell J, Appel C, et al. A global database of COVID-19 vaccinations. Nature Human Behaviour. 2021;5(7):947–953. doi:10.1038/s41562-021-01122-8.

[4] McBryde E, Meehan M T , Ragonnet R, Jayasundara P , Adekunle A I , Kuddus M A , et al.. COVID-19 vaccination modelling results; 2021. Available from: https://covid-19-aithm.shinyapps.io/vaccine_coverage_analysis/.

[5] MRC Centre for Global Infectious Disease Analysis. Covid-19 Scenario Analysis Tool; 2021. Available from: https://covidsim.org/v6.20210915/?place=us.

[6] MRC Centre for Global Infectious Disease Analysis. Imperial College COVID-19 LMIC Reports; 2020. Available from: https://mrc-ide.github.io/global-lmic-reports/.

[7] Matrajt L, Eaton J, Leung T, Brown ER. Vaccine optimization for COVID-19: Who to vaccinate first? Science Advances. 2020;7(6):eabf1374. doi:10.1101/2020.08.14.20175257.

[8] Matrajt L, Eaton J, Leung T, Dimitrov D, Schiffer JT, Swan DA, et al. Optimizing vaccine allocation for COVID-19 vaccines shows the potential role of single-dose vaccination. Nature Communications. 2021;12(1):3449. doi:10.1038/s41467-021-23761-1.

[9] Prem K, Zandvoort Kv, Klepac P, Eggo RM, Davies NG, for the Mathematical Modelling of Infectious Diseases COVID-19 Working Group C, et al. Projecting contact matrices in 177 geographical regions: An update and comparison with empirical data for the COVID-19 era. PLOS Computational Biology. 2021;17(7):1–19. doi:10.1371/journal.pcbi.1009098.

[10] Halloran ME, Struchiner CJ, Longini IM. Study designs for evaluating different efficacy and effectiveness aspects of vaccines. American Journal of Epidemiology. 1997;146(10):789–803.

[11] Cooper A, Reimann R, Cronin D. About Face 3: The Essentials of Interaction Design. John Wiley & Sons; 2012.

[12] You E. Vue.js - The Progressive JavaScript Framework | Vue.js; 2023. Available from: https://vuejs.org/.

[13] Pallets. Welcome to Flask — Flask Documentation (2.3.x); 2023. Available from: https://flask.palletsprojects.com/en/2.3.x/.

[14] Buckner JH, Chowell G, Springborn MR. Optimal Dynamic Prioritization of Scarce COVID-19 Vaccines. medRxiv. 2020;doi:10.1101/2020.09.22.20199174.

[15] Kerr CC, Stuart RM, Mistry D, Abeysuriya RG, Rosenfeld K, Hart GR, et al. Covasim: An agent-based model of COVID-19 dynamics and interventions. PLOS Computational Biology. 2021;17(7):1–32. doi:10.1371/journal.pcbi.1009149.

[16] Morville P, Rosenfeld L. Information Architecture for the World Wide Web: Designing Large-Scale Web Sites. "O'Reilly Media, Inc."; 2006.

[17] Snyder C. Paper Prototyping: The Fast and Easy Way to Design and Refine User Interfaces. Morgan Kaufmann; 2003.

[18] Moore S, Hill EM, Dyson L, Tildesley MJ, Keeling MJ. Retrospectively modeling the effects of increased global vaccine sharing on the COVID-19 pandemic. Nature Medicine. 2022;28(11):2416–2423. doi:10.1038/s41591-022-02064-y.

[19] Barber RM, Sorensen RJD, Pigott DM, Bisignano C, Carter A, Amlag JO, et al. Estimating global, regional, and national daily and cumulative infections with SARS-CoV-2 through Nov 14, 2021: a statistical analysis. The Lancet. 2022;399(10344):2351–2380. doi:10.1016/S0140-6736(22)00484-6.

[20] Institute for Health Metrics Evaluation. COVID-19 vaccine efficacy summary; 2022. https://www.healthdata.org/covid/covid-19-vaccine-efficacy-summary.

[21] OurWorldInData org. COVID-19 vaccine doses, people with at least one dose, people fully vaccinated, and boosters per 100 people; 2021. https://ourworldindata.org/explorers/coronavirus-data-explorer?zoomToSelection=true&time=2020-03-01..latest&uniformYAxis=0&pickerSort=asc&pickerMetric=location&Metric=Vaccine+doses%2C+people+vaccinated%2C+and+booster+doses&Interval=7-day+rolling+average&Relative+to+Population=false&Color+by+test+positivity=false&country=USA~GBR~CAN~DEU~ITA~IND.

[22] USAID. Haiti Health Workforce Data Audits Lead to Human Resources for Health Improvements;. https://www.hfgproject.org/health-workforce-data-audits-lead-improvements-human-resources-health-[illegible]
#:~:text=The%20nation%20of%20over%2010.9,private%20health%20sector%5Biv%5D.

[23] UNICEF. COVID-19 Market Dashboard;. https://www.unicef.org/supply/covid-19-market-dashboard.

[24] United Nations DoE, Division SAP. World Population Prospects: Total population (both sexes combined) by five-year age group; 2019. Available from: https://population.un.org/wpp/Download/Standard/Population/ [cited April 8, 2022].

[25] Mehrotra DV, Janes HE, Fleming TR, Annunziato PW, Neuzil KM, Carpp LN, et al. Clinical Endpoints for Evaluating Efficacy in COVID-19 Vaccine Trials. Annals of Internal Medicine. 2020;doi:10.7326/M20-6169.

[26] Ferguson NM, Laydon D, Nedjati-Gilani G, Imai N, Ainslie K, Baguelin M, et al. Impact of non-pharmaceutical interventions (NPIs) to reduce COVID-19 mortality and healthcare demand. 2020;.

[27] Zhang J, Litvinova M, Wang W, Wang Y, Deng X, Chen X, et al. Evolving epidemiology and transmission dynamics of coronavirus disease 2019 outside Hubei province, China: a descriptive and modelling study. The Lancet Infectious Diseases. 2020;20(7):793–802. doi:10.1016/S1473-3099(20)30230-9.

[28] Du Z, Wang L, Xu X, Wu Y, Cowling BJ, Meyers LA. The serial interval of COVID-19 from publicly reported confirmed cases. Emerging Infectious Diseases. 2020;26(6):1341–1343.

[29] Bi Q, Lessler J, Eckerle I, Lauer SA, Kaiser L, Vuilleumier N, et al. Insights into household transmission of SARS-CoV-2 from a population-based serological survey. Nat Commun. 2021;12(1):3643. doi:10.1038/s41467-021-23733-5.

[30] CDC. COVID-19 Pandemic Planning Scenarios;. Available from: https://www.cdc.gov/coronavirus/2019-ncov/hcp/planning-scenarios.html.

[31] Lauer SA, Grantz KH, Bi Q, Jones FK, Zheng Q, Meredith HR, et al. The incubation period of coronavirus disease 2019 (COVID-19) from publicly reported confirmed cases: Estimation and application. Annals of Internal Medicine. 2020;doi:10.7326/M20-0504.

[32] Zhang J, Litvinova M, Liang Y, Wang Y, Wang W, Zhao S, et al. Changes in contact patterns shape the dynamics of the COVID-19 outbreak in China. Science. 2020;368(6498):1481–1486.

[33] Wei WE, Li Z, Chiew CJ, Yong SE, Toh MP, Lee VJ. Presymptomatic Transmission of SARS-CoV-2-Singapore. Morbidity and Mortality Weekly Report. 2020;69(14):411–415.

[34] Slifka MK, Gao L. Is presymptomatic spread a major contributor to COVID-19 transmission? Nature Medicine. 2020;26(10):1531–1533. doi:10.1038/s41591-020-1046-6.

[35] Bobrovitz N, Ware H, Ma X, Li Z, Hosseini R, Cao C, et al. Protective effectiveness of previous SARS-CoV-2 infection and hybrid immunity against the omicron variant and severe disease: a systematic review and meta-regression. The Lancet Infectious Diseases;doi:10.1016/S1473-3099(22)00801-5.

[36] Altarawneh HN, Chemaitelly H, Hasan MR, Ayoub HH, Qassim S, AlMukdad S, et al. Protection against the Omicron Variant from Previous SARS-CoV-2 Infection. New England Journal of Medicine. 2022;386(13):1288–1290. doi:10.1056/NEJMc2200133.

[37] Feikin DR, Higdon MM, Abu-Raddad LJ, Andrews N, Araos R, Goldberg Y, et al. Duration of effectiveness of vaccines against SARS-CoV-2 infection and COVID-19 disease: results of a systematic review and meta-regression. The Lancet. 2022;399(10328):924–944. doi:https://doi.org/10.1016/S0140-6736(22)00152-0.

[38] Chemaitelly H, Nagelkerke N, Ayoub HH, Coyle P, Tang P, Yassine HM, et al. Duration of immune protection of SARS-CoV-2 natural infection against reinfection. Journal of Travel Medicine. 2022;29(8). doi:10.1093/jtm/taac109.

Supplemental Material

Model Equations

Unvaccinated:

$$
\begin{aligned}
\frac{dS_i}{dt} &= -\lambda_{S_i}(t) - \sum_{j=1}^{3} \theta_{S_i toSV_{ij}}(t) \qquad (1)\\
\frac{dE_i}{dt} &= \lambda_{S_i}(t) - \gamma_E E_i\\
\frac{dA_i}{dt} &= (1-k_i)\gamma_E E_i - \gamma_A A_i\\
\frac{dP_i}{dt} &= k_i \gamma_E E_i - \gamma_P P_i\\
\frac{dI_i}{dt} &= \gamma_P P_i - (1-h_i)\gamma_I I_i - h_i \sigma I_i\\
\frac{dH_i}{dt} &= h_i \sigma I_i - \gamma_H H_i\\
\frac{dR_i}{dt} &= (1-h_i)\gamma_I I_i - 2\gamma_R R_i - \sum_{j=1}^{3} \theta_{R_i toRV_{ij}}(t)\\
\frac{dRA_i}{dt} &= \gamma_A A_i - 2\gamma_{RA} RA_i - \sum_{j=1}^{3} \theta_{RA_i toRAV_{ij}}(t)\\
\frac{dRH_i}{dt} &= \gamma_H H_i - 2\gamma_{RH} RH_i - \sum_{j=1}^{3} \theta_{RH_i toRHV_{ij}}(t)\\
\frac{dRR_i}{dt} &= 2\gamma_R R_i - 2\gamma_R RR_i - \sum_{j=1}^{3} \theta_{RR_i toRRV_{ij}}(t)\\
\frac{dRRA_i}{dt} &= 2\gamma_{RA} RA_i - 2\gamma_{RA} RRA_i - \sum_{j=1}^{3} \theta_{RRA_i toRRAV_{ij}}(t)\\
\frac{dRRH_i}{dt} &= 2\gamma_{RH} RH_i - 2\gamma_{RH} RRH_i - \sum_{j=1}^{3} \theta_{RRH_i toRRHV_{ij}}(t)
\end{aligned}
$$

Unvaccinated with partial protection acquired from at least one infection:

$$
\begin{aligned}
\frac{dS_{P_i}}{dt} &= 2\gamma_R RR_i + 2\gamma_{RA} RRA_i + 2\gamma_{RH} RRH_i + 2\gamma_{RP} RR_{P_i} + 2\gamma_{RAP} RRA_{P_i} \\
&\quad + 2\gamma_{RHP} RRH_{P_i} - (1 - VE_{SUSP})\lambda_{S_{P_i}}(t) - \sum_{j=1}^{3} \theta_{SP_i toSV_{ij}}(t) \\
\frac{dE_{P_i}}{dt} &= (1 - VE_{SUSP})\lambda_{S_{P_i}}(t) - \gamma_E E_{P_i} \\
\frac{dA_{P_i}}{dt} &= (1 - (1 - VE_{SYMPP})k_i)\gamma_E E_{P_i} - \gamma_A A_{P_i} \\
\frac{dP_{P_i}}{dt} &= (1 - VE_{SYMPP})k_i \gamma_E E_{P_i} - \gamma_P P_{P_i} \\
\frac{dI_{P_i}}{dt} &= \gamma_P P_{P_i} - (1 - (1 - VE_{HP})h_i)\gamma_I I_{P_i} - (1 - VE_{HP})h_i \sigma I_{P_i} \\
\frac{dH_{P_i}}{dt} &= (1 - VE_{HP})h_i \sigma I_{P_i} - \gamma_H H_{P_i} \\
\frac{dR_{P_i}}{dt} &= (1 - (1 - VE_{HP})h_i)\gamma_I I_{P_i} - 2\gamma_{RP} R_{P_i} - \sum_{j=1}^{3} \theta_{RP_i toRB_{ij}}(t) \\
\frac{dRA_{P_i}}{dt} &= \gamma_A A_{P_i} - 2\gamma_{RAP} RA_{P_i} - \sum_{j=1}^{3} \theta_{RAP_i toRAB_{ij}}(t) \\
\frac{dRH_{P_i}}{dt} &= \gamma_H H_{P_i} - 2\gamma_{RHP} RH_{P_i} - \sum_{j=1}^{3} \theta_{RHP_i toRHB_{ij}}(t) \\
\frac{dRR_{P_i}}{dt} &= 2\gamma_{RP} R_{P_i} - 2\gamma_{RP} RR_{P_i} - \sum_{j=1}^{3} \theta_{RRP_i toRRB_{ij}}(t) \\
\frac{dRRA_{P_i}}{dt} &= 2\gamma_{RAP} RA_{P_i} - 2\gamma_{RAP} RRA_{P_i} - \sum_{j=1}^{3} \theta_{RRAP_i toRRAB_{ij}}(t) \\
\frac{dRRH_{P_i}}{dt} &= 2\gamma_{RHP} RH_{P_i} - 2\gamma_{RHP} RRH_{P_i} - \sum_{j=1}^{3} \theta_{RRHP_i toRRHB_{ij}}(t)
\end{aligned}
\tag{2}
$$

Vaccinated with primary series, without distinguishing the vaccine products, and with partial protection provided from vaccines and infection:

$$
\begin{aligned}
\frac{dS_{W_i}}{dt} &= 2\gamma_{RW} RR_{W_i} + 2\gamma_{RAW} RRA_{W_i} + 2\gamma_{RHW} RRH_{W_i} + \sum_{j=1}^{3} \gamma_{SV_j} S_{V_{ij}} \\
&\quad - (1 - VE_{SUSW})\lambda_{S_{W_i}}(t) - \sum_{k=1}^{3}\sum_{j=1}^{3} \theta_{SW_{i,V_k} toSB_{ij}}(t) \\
\frac{dE_{W_i}}{dt} &= (1 - VE_{SUSW})\lambda_{S_{W_i}}(t) - \gamma_E E_{W_i} \\
\frac{dA_{W_i}}{dt} &= (1 - (1 - VE_{SYMPW})k_i)\gamma_E E_{W_i} - \gamma_A A_{W_i} \\
\frac{dP_{W_i}}{dt} &= (1 - VE_{SYMPW})k_i \gamma_E E_{W_i} - \gamma_P P_{W_i} \\
\frac{dI_{W_i}}{dt} &= \gamma_P P_{W_i} - (1 - (1 - VE_{HW})h_i)\gamma_I I_{W_i} - (1 - VE_{HW})h_i \sigma I_{W_i} \\
\frac{dH_{W_i}}{dt} &= (1 - VE_{HW})h_i \sigma I_{W_i} - \gamma_H H_{W_i} \\
\frac{dR_{W_i}}{dt} &= (1 - (1 - VE_{HW})h_i)\gamma_I I_{W_i} - 2\gamma_{RW} R_{W_i} - \sum_{k=1}^{3}\sum_{j=1}^{3} \theta_{RW_{i,V_k} toRB_{ij}}(t) \\
\frac{dRA_{W_i}}{dt} &= \gamma_A A_{W_i} - 2\gamma_{RAW} RA_{W_i} - \sum_{k=1}^{3}\sum_{j=1}^{3} \theta_{RAW_{i,V_k} toRAB_{ij}}(t) \\
\frac{dRH_{W_i}}{dt} &= \gamma_H H_{W_i} - 2\gamma_{RHW} RH_{W_i} - \sum_{k=1}^{3}\sum_{j=1}^{3} \theta_{RHW_{i,V_k} toRHB_{ij}}(t) \\
\frac{dRR_{W_i}}{dt} &= 2\gamma_{RW} R_{W_i} - 2\gamma_{RW} RR_{W_i} - \sum_{k=1}^{3}\sum_{j=1}^{3} \theta_{RRW_{i,V_k} toRRB_{ij}}(t) \\
\frac{dRRA_{W_i}}{dt} &= 2\gamma_{RAW} RA_{W_i} - 2\gamma_{RAW} RRA_{W_i} - \sum_{k=1}^{3}\sum_{j=1}^{3} \theta_{RRAW_{i,V_k} toRRAB_{ij}}(t) \\
\frac{dRRH_{W_i}}{dt} &= 2\gamma_{RHW} RH_{W_i} - 2\gamma_{RHW} RRH_{W_i} - \sum_{k=1}^{3}\sum_{j=1}^{3} \theta_{RRHW_{i,V_k} toRRHB_{ij}}(t)
\end{aligned} \tag{3}
$$

Vaccinated with primary series and vaccine product j:

$$
\begin{aligned}
\frac{dS_{V_{ij}}}{dt} &= \theta_{S_i toSV_{ij}}(t) + \theta_{SP_i toSV_{ij}}(t) + \gamma_{SB_j} S_{B_{ij}} - \sum_{k=1}^{3} \theta_{SV_{ij} toSB_{ik}}(t) \\
&\quad - (1 - VE_{SUSV_j})\lambda_{S_{V_{ij}}}(t) - \gamma_{SV_j} S_{V_{ij}} \\
\frac{dE_{V_{ij}}}{dt} &= (1 - VE_{SUSV_j})\lambda_{S_{V_{ij}}}(t) - \gamma_E E_{V_{ij}} \\
\frac{dA_{V_{ij}}}{dt} &= (1 - (1 - VE_{SYMPV_j})k_i)\gamma_E E_{V_{ij}} - \gamma_A A_{V_{ij}} \\
\frac{dP_{V_{ij}}}{dt} &= (1 - VE_{SYMPV_j})k_i \gamma_E E_{V_{ij}} - \gamma_P P_{V_{ij}} \\
\frac{dI_{V_{ij}}}{dt} &= \gamma_P P_{V_{ij}} - (1 - (1 - VE_{HV_j})h_i)\gamma_I I_{V_{ij}} - (1 - VE_{HV_j})h_i \sigma I_{V_{ij}} \\
\frac{dH_{V_{ij}}}{dt} &= (1 - VE_{HV_j})h_i \sigma I_{V_{ij}} - \gamma_H H_{V_{ij}} \\
\frac{dR_{V_{ij}}}{dt} &= (1 - (1 - VE_{HV_j})h_i)\gamma_I I_{V_{ij}} - 2\gamma_{RV_j} R_{V_{ij}} - \sum_{k=1}^{3} \theta_{RV_{ij} toRB_{ik}}(t) \\
\frac{dRA_{V_{ij}}}{dt} &= \gamma_A A_{V_{ij}} - 2\gamma_{RAV_{ij}} RA_{V_{ij}} - \sum_{k=1}^{3} \theta_{RAV_{ij} toRAB_{ik}}(t) \\
\frac{dRH_{V_{ij}}}{dt} &= \gamma_H H_{V_{ij}} - 2\gamma_{RHV_{ij}} RH_{V_{ij}} - \sum_{k=1}^{3} \theta_{RHV_{ij} toRHB_{ik}}(t) \\
\frac{dRR_{V_{ij}}}{dt} &= 2\gamma_{RV_j} R_{V_{ij}} - 2\gamma_{RV_j} RR_{V_{ij}} - \sum_{k=1}^{3} \theta_{RRV_{ij} toRRB_{ik}}(t) \\
\frac{dRRA_{V_{ij}}}{dt} &= 2\gamma_{RAV_j} RA_{V_{ij}} - 2\gamma_{RAV_j} RRA_{V_{ij}} - \sum_{k=1}^{3} \theta_{RRAV_{ij} toRRAB_{ik}}(t) \\
\frac{dRRH_{V_{ij}}}{dt} &= 2\gamma_{RHV_j} RH_{V_{ij}} - 2\gamma_{RHV_j} RRH_{V_{ij}} - \sum_{k=1}^{3} \theta_{RRHV_{ij} toRRHB_{ik}}(t)
\end{aligned} \tag{4}
$$

Vaccinated and boosted with vaccine product j:

$$
\begin{aligned}
\frac{dS_{B_{ij}}}{dt} &= \sum_{k=1}^{3} \theta_{SV_{ik}toSB_{ij}}(t) + \sum_{k=1}^{3} \theta_{SW_{i,V_k}toSB_{ij}}(t) + 2\gamma_{RV_j} RR_{V_{ij}} + 2\gamma_{RAV_j} RRA_{V_{ij}} \\
&+ 2\gamma_{RHV_j} RRH_{V_{ij}} + 2\gamma_{RB_j} RR_{B_{ij}} + 2\gamma_{RAB_j} RRA_{B_{ij}} + 2\gamma_{RHB_j} RRH_{B_{ij}} \\
&- (1 - VE_{SUSB_j})\lambda_{S_{B_{ij}}}(t) - \gamma_{SB_j} S_{B_{ij}} \\
\frac{dE_{B_{ij}}}{dt} &= (1 - VE_{SUSB_j})\lambda_{S_{B_{ij}}}(t) - \gamma_E E_{B_{ij}} \\
\frac{dA_{B_{ij}}}{dt} &= (1 - (1 - VE_{SYMPB_j}))k_i \gamma_E E_{B_{ij}} - \gamma_A A_{B_{ij}} \\
\frac{dP_{B_{ij}}}{dt} &= (1 - VE_{SYMPB_j})k_i \gamma_E E_{B_{ij}} - \gamma_P P_{B_{ij}} \\
\frac{dI_{B_{ij}}}{dt} &= \gamma_P P_{B_{ij}} - (1 - (1 - VE_{HB_j}))h_i \gamma_I I_{B_{ij}} - (1 - VE_{HB_j})h_i \sigma I_{B_{ij}} \\
\frac{dH_{B_{ij}}}{dt} &= (1 - VE_{HB_j})h_i \sigma I_{B_{ij}} - \gamma_H H_{B_{ij}} \\
\frac{dR_{B_{ij}}}{dt} &= (1 - (1 - VE_{HB_j}))h_i \gamma_I I_{B_{ij}} + \theta_{RP_itoRB_{ij}}(t) + \sum_{k=1}^{3} \theta_{RW_{i,V_k}toRB_{ij}}(t) \\
&+ \sum_{k=1}^{3} \theta_{RV_{ik}toRB_{ij}}(t) - 2\gamma_{RB_j} R_{B_{ij}} \\
\frac{dRA_{B_{ij}}}{dt} &= \gamma_A A_{B_{ij}} + \theta_{RAP_itoRAB_{ij}}(t) + \sum_{k=1}^{3} \theta_{RAW_{i,V_k}toRAB_{ij}}(t) + \sum_{k=1}^{3} \theta_{RAV_{ik}toRAB_{ij}}(t) \\
&- 2\gamma_{RAB_j} RA_{B_{ij}} \\
\frac{dRH_{B_{ij}}}{dt} &= \gamma_H H_{B_{ij}} + \theta_{RHP_itoRHB_{ij}}(t) + \sum_{k=1}^{3} \theta_{RHW_{i,V_k}toRHB_{ij}}(t) + \sum_{k}^{3} \theta_{RHV_{ik}toRHB_{ij}}(t) \\
&- 2\gamma_{RHB_j} RH_{B_{ij}} \\
\frac{dRR_{B_{ij}}}{dt} &= 2\gamma_{RB_j} R_{B_{ij}} + \theta_{RRP_itoRRB_{ij}}(t) + \sum_{k=1}^{3} \theta_{RRW_{i,V_k}toRRB_{ij}}(t) + \sum_{k=1}^{3} \theta_{RRV_{ik}toRRB_{ij}}(t) \\
&- 2\gamma_{RB_j} RR_{B_{ij}} \\
\frac{dRRA_{B_{ij}}}{dt} &= 2\gamma_{RAB_j} RA_{B_{ij}} + \theta_{RRAP_itoRRAB_{ij}}(t) + \sum_{k=1}^{3} \theta_{RRAW_{i,V_k}toRRAB_{ij}}(t) \\
&+ \sum_{k=1}^{3} \theta_{RRAV_{ik}toRRAB_{ij}}(t) - 2\gamma_{RA_{B_j}} RRA_{B_{ij}} \\
\frac{dRRH_{B_{ij}}}{dt} &= 2\gamma_{RH_{B_j}} RH_{B_{ij}} + \theta_{RRHP_itoRRHB_{ij}}(t) + \sum_{k=1}^{3} \theta_{RRHW_{i,V_k}toRRHB_{ij}}(t) \\
&+ \sum_{k=1}^{3} \theta_{RRHV_{ik}toRRHB_{ij}}(t) - 2\gamma_{RH_{B_j}} RRH_{B_{ij}}
\end{aligned} \tag{5}
$$

The forces of infection for each compartment, $\alpha = i, P_i, W_i, V_{ij}, B_{ij}$, $i = 1, 2, \dots 5$ and $j = 1, 2, 3$, is:

$$\lambda_{S_\alpha}(t) = m_i \lambda_i S_\alpha,$$

where

$$\lambda_i = \beta \sum_{\alpha \in \{k, P_k, W_k, V_{kj}, B_{kj}\}_{k=1,\dots,5, j=1,2,3}} \frac{C_{i,k}(r_A A_\alpha + r_P P_\alpha + r_H H_\alpha + I_\alpha)}{N_k},$$

where N_k is the population size of each age-group k, d_k is the social distancing weights, and C is the contact matrix, $k = 1, 2, \dots, 5$.

Vaccination Rate. We denote with $V_{ij}(t)$ the number of vaccines of vaccine product j to allocate per day among the available individuals $X_i(t)$ from age-group i at time $t \geq 0$, $i = 1, 2, \dots, 5$, $j = 1, 2, 3$. The vaccination rate at time t is

$$\theta_i(t) = \theta_{X_i to X V_{ij}}(t) = \begin{cases} 0 & \text{if } t > t^* \\ \min\{X_i(t), V_{ij}(t)\} & \text{if } 0 \leq t \leq t^*, \end{cases} \quad (6)$$

where t^* is the ending time of the vaccination campaign with vaccine type j.

Data sources and model default parameters

Population structure: We use contact matrices for regions given in [9]. The contact matrices for the following regions: Australia, Haiti, Japan, Lebanon, Somalia, Taiwan were imputed by assuming similar contacts as the neighboring countries (New Zealand, Dominican Republic, South Korea, Syria, Djibouti and South Korea respectively). For each region, the total population and age distribution were obtained from [24], with population densities correspond to the 2022 estimates.

Vaccine effectiveness: Vaccine effectiveness parameters were based on the data presented in [19]. We assume that there is a multiplicative relationship between the vaccine efficacy against laboratory-confirmed COVID-19 disease, VE_{DIS}, VE_{SUS} and VE_{SYMP} [25], so that

$$\text{VE}_{\text{DIS}} = 1 - (1 - \text{VE}_{\text{SUS}})(1 - \text{VE}_{\text{SYMP}}). \quad (7)$$

And similarly,

$$\text{VE}_{\text{SEV}} = 1 - (1 - \text{VE}_{\text{H}})(1 - \text{VE}_{\text{SYMP}}). \quad (8)$$

Using this relationship and early vaccine effectiveness estimates, we derived VE_{SYMP} and VE_{H} from given estimates for VE_{DIS} and VE_{SEV}. Furthermore, when vaccine effectiveness estimates were not available for Omicron, we imputed them in the following way: we computed the reduction in the effectiveness between ancestral strain and Omicron for those vaccine products for which the data was available, and use that as a multiplier for those products for which we could not find an estimate. Table summarizes the default values for vaccine effectiveness for each vaccine product provided.

	Vaccine effectiveness					
	Omicron (Primary series)			Omicron (booster)		
Vaccine	Hospitalization VE_H	Infection VE_{SUS}	Symptomatic infection VE_{SYMP}	Hospitalization VE_H	Infection VE_{SUS}	Symptomatic infection VE_{SYMP}
AstraZeneca	0.71	0.36	0.29	0.94	0.63	0.63
Cansino	0.48	0.32	0.26	0.66	0.62	0.66
CoronaVac	0.37	0.24	0.26	0.5	0.47	0.65
Covaxin	0.57	0.38	0.31	0.78	0.73	0.78
Jansen	0.57	0.33	0.26	0.86	0.72	0.67
Moderna	0.73	0.48	0.36	0.97	0.92	0.92
Novavax	0.65	0.43	0.36	0.89	0.83	0.90
Pfizer/BioNTech	0.72	0.44	0.30	0.95	0.86	0.88
Vero Cell	0.53	0.35	0.31	0.73	0.68	0.78
Sputnik-V	0.67	0.44	0.36	0.92	0.86	0.92

Natural history Parameters: Hospitalization and mortality rates were obtained from [26]. Because the rates in [26] are given for different age groups than the ones we considered, we adjusted them to each region's population in agreement with that region's population composition.

Tables S1 and S2 provide the default parameters provided in **Covid19Vaxplorer**. However, the user can change all these parameters to match their particular location and experince.

Parameter	Value	Reference
**SARS-CoV-2 infection and natural history parameters**		
Average time between symptom onset and hospitalization	3.8	[27]
Mean Duration of Infectiousness after developing symptoms	4	[28, 29]
Mean duration of hospitalization	7	[30]
Mean duration of latent period	2	[31, 32]
Mean duration of pre-symptomatic period	1.5	[33]
Relative infectiousness of asymptomatic infected individuals	1	[30]
Relative infectiousness of hospitalized infected individuals	0	Assumed
Relative infectiousness of pre-symptomatic infected individuals	1	[34]
Mean duration of immunity after SARS-CoV-2 infection (first phase)	100	[35]
Mean duration of immunity after SARS-CoV-2 infection (second phase)	180	[36]
Mean duration of immunity for waned vaccinated individuals following a SARS-CoV-2 infection	100	[35]
Mean duration of vaccine-induced immunity for vaccination with a primary series	100	[35]
Mean duration of immunity for vaccinated individuals with a primary series following a SARS-CoV-2 infection (hybrid immunity)	180	[35]
Mean duration of vaccine-induced immunity for vaccination with a booster	180	[37, 38]
Mean duration of immunity for vaccinated individuals with a booster following a SARS-CoV-2 infection (hybrid immunity)	180	[37]

Table S1: Description of parameters used in the model.

Parameter	Value	Reference
**Protection parameters for partially susceptible individuals**		
Protection against infection for partially susceptible individuals	0	[35]
Protection against symptomatic infection for partially susceptible individuals	24.7	[35]
Protection against hospitalization for partially susceptible individuals	74.6	[35]
**Protection parameters for partially vaccinated individuals**		
Protection against infection for partially susceptible vaccinated individuals	0	[35]
Protection against symptomatic infection for partially susceptible vaccinated individuals	41	[35]
Protection against hospitalization for partially susceptible vaccinated individuals	95.3	[35]

Table S2: Description of parameters used in the model.

**Vaccine**	**Group 1 0-19**	**Group 2 20-49**	**Group 3 50-64**	**Group 4 65-74**	**Group 5 75+**
Covishield	0	0	0	0	250,000
Unknown	0	0	0	56,154	23,929
Sinopharm	0	0	648,845	701,155	0
AstraZeneca	0	0	210,000	0	0
Covaxin	0	0	250,000	0	0
Jansen	0	7,270,765	1,343,991	0	0
**Total**	**0**	**7,270,765.000**	**2,452,836.000**	**757,309.000**	**273,929.000**

Table S3: Assumed distribution of COVID-19 prior vaccination in Afghanistan per age group and vaccine product.

Supplemental Figures

Strategy 1: Vaccinate as many people with boosters as possible.

Vaccine 1: Jansen vaccine

Table 1: Percent of Jansen vaccine given as primary series

	G1:0-19	G2:20-49	G3:50-64	G4: 65-74	G5:75+
Jansen	0%	0%	39.49%	37.02%	0%

Table 2: Percent of Jansen vaccine given as boosters

	G1:0-19	G2:20-49	G3:50-64	G4: 65-74	G5:75+
Jansen	0%	1.23%	0.27%	1.16%	20.83%
Convidecia	0%	0%	0%	0%	0%
Vero Cell	0%	0%	0%	0%	0%

Vaccine 2: Convidecia vaccine

Table 3: Percent of Convidecia vaccine given as primary series

	G1:0-19	G2:20-49	G3:50-64	G4: 65-74	G5:75+
Convidecia	0.00%	3.33%	45.66%	0%	0%

Table 4: Percent of Convidecia vaccine given as boosters

	G1:0-19	G2:20-49	G3:50-64	G4: 65-74	G5:75+
Jansen	0%	0%	26.33%	24.68%	0%
Convidecia	0%	0%	0%	0%	0%
Vero Cell	0%	0%	0%	0%	0%

Vaccine 3: Vero Cell vaccine

Table 5: Percent of Vero Cell vaccine given as primary series

	G1:0-19	G2:20-49	G3:50-64	G4: 65-74	G5:75+
Vero Cell	0.00%	26.51%	0.00%	0.00%	0.00%

Table 6: Percent of Vero Cell vaccine given as boosters

	G1:0-19	G2:20-49	G3:50-64	G4: 65-74	G5:75+
Jansen	0%	0%	0%	0%	0%
Convidecia	0%	5.00%	68.49%	0%	0%
Vero Cell	0%	0%	0%	0%	0%

Strategy 2: Vaccinate as many people with primary series as possible.

Vaccine 1: Jansen vaccine

Table 1: Percent of Jansen vaccine given as primary series

	G1:0-19	G2:20-49	G3:50-64	G4: 65-74	G5:75+
Jansen	0.0%	0.0%	0.0%	100.0%	0.0%

Table 2: Percent of Jansen vaccine given as boosters

	G1:0-19	G2:20-49	G3:50-64	G4: 65-74	G5:75+
Jansen	0%	0%	0%	0%	0%
Convidecia	0%	0%	0%	0%	0%
Vero Cell	0%	0%	0%	0%	0%

Vaccine 2: Convidecia vaccine

Table 3: Percent of Convidecia vaccine given as primary series

	G1:0-19	G2:20-49	G3:50-64	G4: 65-74	G5:75+
Convidecia	0.0%	70.00%	30.00%	0.0%	0.0%

Table 4: Percent of Convidecia vaccine given as boosters

	G1:0-19	G2:20-49	G3:50-64	G4: 65-74	G5:75+
Jansen	0%	0%	0%	0%	0%
Convidecia	0%	0%	0%	0%	0%
Vero Cell	0%	0%	0%	0%	0%

Vaccine 3: Vero cell vaccine

Table 5: Percent of Vero Cell vaccine given as primary series

	G1:0-19	G2:20-49	G3:50-64	G4: 65-74	G5:75+
Vero Cell	0.0%	26.5%	0.0%	0.0%	0.0%

Table 6: Percent of Vero Cell vaccine given as boosters

	G1:0-19	G2:20-49	G3:50-64	G4: 65-74	G5:75+
Jansen	0%	0%	0%	0%	0%
Convidecia	0%	0%	0%	0%	0%
Vero Cell	0%	0%	0%	0%	0%

Figure S1: Tables with the distributions across age groups of Jansen, Convidecia and Vero Cell vaccines to be given as a primary series or as boosters under the strategies of **“Boosters first” (Strategy 1)** and **“Primary series first” (Strategy 2)** for the hypothetical example in Haiti.

Strategy 1: Vaccinate as many people with boosters as possible.

Vaccine 3: Jansen vaccine

Table 1: Percent of Jansen vaccine given as primary series

	G1:0-19	G2:20-49	G3:50-64	G4: 65-74	G5:75+
Vaccine 3: Jansen	0%	0%	0%	0%	0%

Table 2: Percent of Jansen vaccine given as boosters

	G1:0-19	G2:20-49	G3:50-64	G4: 65-74	G5:75+
Vaccine 1: Vaxzevira	0%	0.00%	3.07%	0.00%	3.66%
Vaccine 2: Sinopharm, Covaxin & Unknown	0%	0%	13%	11%	0%
Vaccine 3: Jansen	0%	49%	20%	0%	0%

Strategy 2: Vaccinate as many people with primary series as possible.

Vaccine 3: Jansen vaccine

Table 1: Percent of Jansen vaccine given as primary series

	G1:0-19	G2:20-49	G3:50-64	G4: 65-74	G5:75+
Vaccine 3: Jansen	0%	100%	0%	0%	0%

Table 2: Percent of Jansen vaccine given as boosters

	G1:0-19	G2:20-49	G3:50-64	G4: 65-74	G5:75+
Vaccine 1: Vaxzevira	0%	0%	0%	0%	0%
Vaccine 2: Sinopharm, Covaxin & Unknown	0%	0%	0%	0%	0%
Vaccine 3: Jansen	0%	0%	0%	0%	0%

Figure S2: Tables with the distributions across age groups of the Jansen vaccine to be given as a primary series or as boosters under the strategies of **"Boosters first" (Strategy 1)** and **"Primary series first" (Strategy 2)** in a hypothetical scenario in Afghanistan.

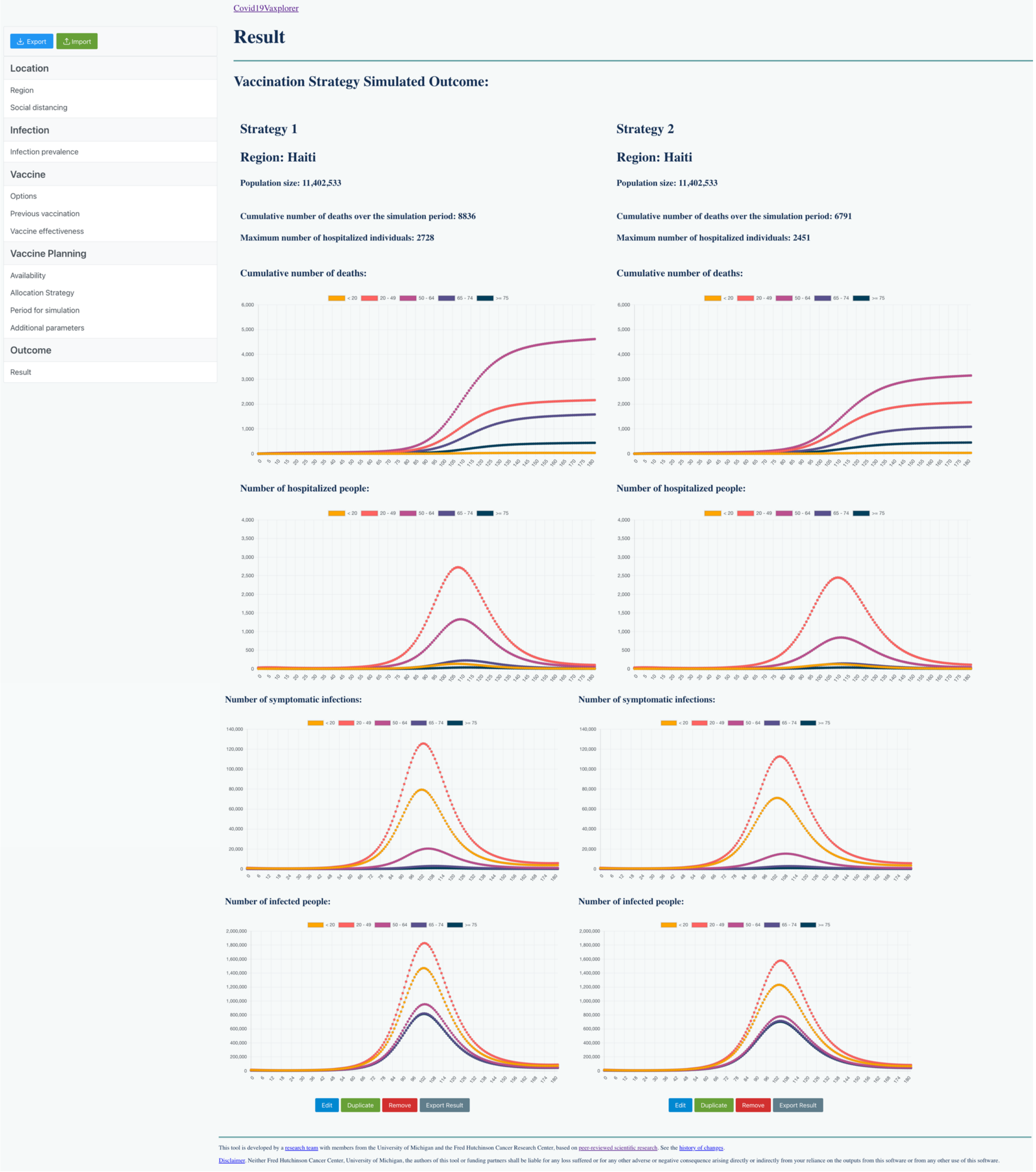

Figure S3: **Covid19Vaxplorer** results page for comparing two strategies in a hypothetical situation in Haiti assuming 80% of the population has pre-existing immunity. Strategy 1, "boosters first" strategy, would result in 8,836 cumulative deaths at the end of the simulation period . In contrast, Strategy 2, "primary doses first" would result in 6791 cumulative deaths.
