# Supplementary material for: Covid19Vaxplorer: A free, online, user-friendly COVID-19 vaccine allocation comparison tool
Source: PLOS Glob Public Health. 2024 Jan 22;4(1):e0002136. doi: 10.1371/journal.pgph.0002136 (PMC10802966; doi:10.1371/journal.pgph.0002136)
Supplement: Attachment — Submitted filename: Covid19VaxplorerRebuttalLetter.pdf [file pgph.0002136.s014.pdf]

Rebuttal letter

We thank the reviewers for their careful reading of our manuscript and their comments. Responses to each comment are given below in blue.

5. Review Comments to the Author

Reviewer #1: The article deals with a very important topic and the goal is worthy. The article should be improved in different ways, especially in the presentation of details. The model and the developed software have a lot of details that need to be explained. The authors probably devoted a lot of time and now they need to give more value and clarity to their work.

The authors must emphasize the aim of the article. For instance, we developed Covid19Vaxplorer in order to …?

We thank the reviewer for their positive comments on our work. We have rewritten several portions of the manuscript in response to these critiques. Further details are given below.

Not clear why the authors mentioned the 183 regions in the abstract. I think there are more important aspects that can be mentioned/clarified in the abstract.

We thank the reviewer for this comment. We have added more details and other aspects to the abstract. We left the number of regions because we believe that it is important to highlight the fact that Covid19Vaxplorer can be used in most parts of the world.

The results section in the abstract starts with “we usage two examples”. I think this section can be improved a lot, mentioning the model, software and the applications of this work.

We agree, we have modified the abstract to add these details.

The introduction must be improved. There are some related works regarding vaccination strategies and mathematical models. The authors should provide a good review of these in order to present a better background to the topic. The current version looks short on this matter.

We thank the reviewer for this comment. We have now added a selection (there are many!) of mathematical modeling papers for vaccination strategies and related work.

The presentation of the model should be clearer. It would be very difficult for many users to understand the model. I suggest adding a Table that describes the different groups in detail and how they differentiate. Figure 1 is useful, but it seems not enough.

We thank the reviewer for this comment. We have expanded the methods section considerably adding subsections that explain each component of the model.

Page 4. Explain better "We model vaccination campaigns with a primary series or boosters. We do not model individual doses for primary series."

This sentence has been modified for clarity.

Page 4. Please explain the rationale to distribute the vaccines proportionally between groups. Make sense that recovered groups have the same rate?

We thank the reviewer for this question. We have significantly expanded the Methods section so that this and the other comments are addressed (see section Vaccine distribution among compartments).

. In brief, there are two levels of vaccine distribution: vaccine distribution among compartments (within a single age and/or vaccinated group) and vaccine distribution among age groups and groups previously vaccinated. The user gets to determine which age groups get vaccinated, and how much vaccine is allocated to them, and they also decide if that vaccine will be used as a primary series or as a booster (if possible). Once they have decided who gets vaccinated and with how much vaccine, we distribute that vaccine among the corresponding compartments in that age group. For example, if vaccine will be used as a primary series, then vaccine will be distributed among all the unvaccinated compartments of that age group except for those who are currently infected and symptomatic.

This rationale is equivalent to assuming that people in the community will get the vaccine randomly. This is a good assumption because individuals who are not currently infected and symptomatic do typically get vaccinated, irrespective of their previous infections. We have added a sentence in the manuscript to explain this rationale.

Page 4. Please explain “Vaccinated individuals from compartments E_, A_, and P_ do no acquire any type of vaccine protection.”

We thank the reviewer for this comment. Along the lines of the comment above, we assume that people in the infected compartments who are not symptomatic might seek

a vaccine, however, the vaccine will have no effect on them. We have clarified this sentence in the manuscript. We have added a whole section in the methods describing how this is done (see section Vaccine distribution among compartments).

Page 4. Typo Vij,, Vij. ??

Yes, thank you for catching that. We have corrected it as Vij, Bij.

Page 6. The authors mentioned: “Third, users are invited to specify planning-related parameters, including vaccine availability (what and when), and how vaccines will be allocated to different age groups within the population.”
Here I am confused since previously the authors mentioned that allocation of vaccines is proportional to the size of the populations. This and other details need much better explanation.

We thank the reviewer for this comment. We have significantly modified the methods section so that hopefully this will be more clear now. Please see response above about how the vaccine is allocated proportionally among compartments. In this particular section, we are referring to the part of the model where the user decides how to distribute vaccine among age groups. which age groups get vaccinated, and how much vaccine is allocated to them, and they also decide if that vaccine will be used as a primary series or as a booster (if possible), see section Vaccine allocation strategy in Methods.

Page 6. The authors stated “Finally, users are presented with projections of key epidemic outcomes based on the information they input.” Please add details about what are these outcomes. It is quite difficult to grasp the aims of the developed software.

Thank you for this comment. We have modified to add more details to the outcomes the text and added a figure for the readers to see what the outcomes are.

Page 7. The authors should explain how the input value of the Ro is used in the model. This is not clear. For instance, the authors mentioned that the duration of the infectious period is chosen by the user. What expression Ro is used? Comment on the fact that Ro is related to one infective person introduced in a fully susceptible population. How does the software handle the Ro as an input? This needs more explanation in the manuscript.

We thank the reviewer for bringing this point. We have added a description of how R0 is used in the model to compute the force of infection and we have added in the supplemental material the full description of the method used.

The authors wrote:
"Social distancing: User selects how people are practicing social distancing at each location (home, work, school, community) if people are practicing social distancing, ranging from "fully social distanced" to "not at all" social distanced. These inputs are translated to the model as multipliers to each of these contact matrices"
This needs more details. For instance, fully social distanced translate on zero transmission rates?. Is not clear how the location (home,work,school,community) is integrated in the model presented in Figure 1.

We thank the reviewer for this comment. We have added a section in the Methods (see Modeling Contacts and Social Distancing) describing how contacts in each location are modeled and how social distancing is modeled.

Authors wrote:
"Infection Prevalence: In this window, the user inputs the proportion of people in each age group who have been previously infected (and who then will have some protection against reinfection, symptoms and/or severe disease) and those who are currently infected. The window provides default percentages for cumulative prior infection as of November 2021 taken from [19]."
In some way these are the initial conditions for the 540 subpopulations. This is a very complex task that need further explanation. Some details about the default values should be added. For instance, as the authors mentioned each country has different percentages of vaccinated people. How the default option takes this into account.

We thank the reviewer for this comment. We have added a section explaining how the initial conditions are set in the model using the information provided by the user or default preexisting prevalence (taken from Barber et al, 2022) if none is given (see section Modeling Initial Conditions in Methods). We do not assume any preexisting default vaccination. If the user does not provide pre-existing vaccination, then we assume that no one has been vaccinated. We have made this explicit in the text. We have included an additional figure in the supplemental material showing the window where this information is input in Covid19Vaxplorer.

Page 8. The authors wrote:
“Period for simulation: The user provides the beginning and end dates for the simulation.
Default end dates are provided based on the amounts of vaccine available and vaccination rates,”
Please explain more details about this process. Assuming a particular number of vaccines and a rate how the end date is computed. Due to the structure of the model (ODEs?) and the way vaccines are assigned this point is not clear.

We thank the reviewer for pointing this out. We have clarified how we obtained the end date for the simulation and for the vaccination campaign and we have provided an example to help with the understandability of the model (see section: Duration of vaccination campaign/simulation). We have added a supplemental figure showing the window in Covid19Vaxplorer to improve the understanding of this point.

Page 8. “4. Outcome: This is where the results are displayed and visualized”
Does the software include the epidemic curves by age groups and for any group? Please add comments on the manuscript.

We have added additional details to the manuscript. It now reads:
“*The following key outcomes are given: Cumulative number of deaths over the simulation period, Maximum number of hospitalized individuals, epidemic curves for the daily number of deaths, hospitalizations, symptomatic infections and infections. Each of these outcomes is displayed by age group. These results can be downloaded as a csv file for additonal analysis.”*

In addition, we have added a reference to the results figure to show the outputs from Covid19Vaxplorer.

End of page 8. The authors wrote:
“For both strategies, vaccination starts with older age groups and goes in decreasing order by age. For example, under in Strategy 1, we would start vaccinating the oldest age group (group 5, 75 years old and older),”
Does always the vaccination strategy start with the oldest age group or is this just for the example on the Results section. Better explanation with more details needed.

We thank the reviewer for this comment. The text was indeed very confusing. We have now clarified the text to make it clear that it is just for the example in this section. We have further added in the Methods section a whole subsection entitled Order of vaccination among selected groups where we clarify that Covid19Vaxplorer always vaccinates all age groups for which vaccine has been allocated simultaneously. For this

particular example, we prioritized older adults in the percentage of vaccine allocated to them, but they will be vaccinated at the same time with all the other groups where vaccine was allocated.

Page 11. The authors wrote:
“then adults in group 3 (50-65 years old) and so on as vaccine availability permits.” Please add detailed explanation. Is not clear if the vaccines can be allocated to different groups and with different proportions at the same time.

We agree with the reviewer. We have modified the text and given more details. Please see comment above.

Page 11. The authors wrote:

“sensitivity analysis with 80% pre-existing immunity showed similar qualitative results with a strategy allocating primary series first outperforming the one prioritizing boosters” Please add more details about all the parameter used in this scenario and add discussion. For instance, outperforming based on what? Number of deaths? number of cases? etc.

We thank the reviewer for this comment. More details have been given to this example.

Page 11. Authors wrote:
“We consider a hypothetical situation in which Haiti would experience a new SARS-CoV-2 wave with a variant with similar infectivity as the delta variant (R0 = 3) with 0.1% of the population currently infected. ” Please explain how this is achieved in the software. It is not clear. The authors mean just assuming that R0=3 from the beginning of the simulation on the authors mean that the new variant is introduced at some point?

Thanks for pointing this out. We assume R0 = 3 from the beginning of the simulation, although it is important to note that this will not be the effective reproductive number, which will be affected by the percentage of the population with previous infections. We have added a sentence in the manuscript to clarify this point.

Page 11. The authors wrote:
“We assume that for all three vaccines, 50,000 individuals can be vaccinated daily.” Please explain how the vaccines are distributed. How the model is modified such 50k vaccines can be allocated.

We thank the reviewer for this comment. The tool has a window where the vaccination rate can be input for each vaccine product. We have explained this in the text and added a supplemental figure to show the usage of the tool.

Page 15. The authors wrote:
"While it incorporates product-specific information about the length of vaccine-induced immunity and its effectiveness, it assumes a single duration of immunity for waned vaccinated individuals".

Not clear why the authors mention that the model assumes a single duration of immunity. Please revise this statement. The waning is modeled by a differential equation with a constant coefficient.

We thank the reviewer for this comment, we have rewritten the methods section and clarified that while the model allows up to three different vaccine products with independent compartments for each product for primary series and boosters (so each vaccine product gives rise to its own set of differential equations) there is a single waned vaccinated class (Figure 1, part c).

Page 15. Typo : "used or construed"

To avoid confusion we have changed the word construed by "interpreted".

In summary, I think the article is valuable and deals with an extremely important topic. The authors need to explain much more details. The presentation of the model should be clearer. It would be very difficult for many users to understand the model. Probably the article needs to be extended to make a better presentation. Not sure if the journal has page limitations. There are many more limitations that the authors need to mention. For instance, the values of the parameters play an important role on the dynamics and they have uncertainty. This is just one example of limitations that can be added. Variants?

We agree, we have expanded the discussion and the limitations section to discuss the limitations further.

Reviewer #2: Review: Covid19Vaxplorer: a free, online, user-friendly COVID-19 Vaccine Allocation Comparison Tool
The manuscript describes a developed online tool proposed for use to assess the impact on different COVID-19 vaccination strategies on covid related outcomes that includes hospitalization and mortality. The authors use deterministic mathematical

models.
Comments
1. This tool would probably have been more useful and relevant in the immediate post availability of COVID-19 vaccines and when the COVID-19 pandemic was still growing with high levels of deaths and hospitalizations. For various reasons vaccination uptake anywhere in the World has dramatically come down, and country priorities on COVID-19 vaccination have probably shifted.

We agree with the reviewer. As with many tools and models developed during the pandemic, the corresponding manuscript and tool came later than expected, and suffered from drawbacks with funding and personnel. We believe however that publishing the software and the companion manuscript is still important for the following reasons: 1. While the reviewer is correct that most countries have different priorities now, it is still true that many low income countries continue to have very low vaccination rates. It is still a priority for these countries to protect their populations, especially given that new variants emerge continuously, and it is plausible that a new variant could evade preexisting immunity. 2. This tool is potentially useful for other respiratory viruses, as the model and the tool can be easily adapted, and it is free and available for download. 3. It might be useful for vaccine planning for other infectious diseases sharing transmission pathways with SARS-CoV-2. 4. As with everything else in science, seeing how this tool was created can help others create their own online tools.

2. Vaccination uptake depends on a number of factors including system factors, which seems what this paper is mostly focusing on, i.e. helping decision makers on vaccination strategies. During the early stages of the COVID-19 epidemic and when vaccines were first introduced, the prohibitive costs of vaccines, vaccine hording where some of the barriers to COVID-19 vaccination roll out in middle- and low-income countries. With COVID-19 vaccines being more readily available and cheaper across the world, the important factors that are most informative of the success of the outcomes of COVID-19 vaccination programmes are likely to be social, cultural, and behavioral factors, and not necessary the systems factors as mostly modelled in this paper.

We fully agree with the reviewer and we thank them for this comment. We have added a paragraph in the discussion pointing this as a limitation of our tool.

3. Although the authors indicated that these models should not be used for predictive purposes, but for comparing different scenarios, it would have been useful to fit the

models to real-life observed scenarios, i.e. test the models on real empirical data to test the reasonableness of the assumptions being made, and how well these models fit to observed experiences.

We agree with the reviewer. There have been extensive mathematical models fit to observed experiences. The aim of this tool was not to fit a model to data, but rather to allow users to compare vaccination strategies. This is mentioned as a limitation of our work.

4. It is not clear from the model if known epidemiological concepts were incorporated in outcomes such as the risk of death and/or serious illness. For example, age was shown to be a risk factor for COVID-19 outcomes, with higher COVID-19 associated mortality and hospitalization being associated with older age. Comorbidities such as hypertension, diabetes, and those with compromised immune systems were also shown to be associated with COVID-19 outcomes.

We thank the reviewer for this comment. The hospitalization and mortality rates are indeed age-stratified. We have added a sentence in the methods to make this more transparent. These rates do not take into account comorbidities, as different countries will have different percentages of comorbidities in their populations. We have added this as a limitation in the Discussion.

5. There are minor comments highlighted on the manuscript.

We thank the reviewer for their comments. They have all been addressed.
