# Supplementary material for: Covid19Vaxplorer: A free, online, user-friendly COVID-19 vaccine allocation comparison tool
Source: PLOS Glob Public Health. 2024 Jan 22;4(1):e0002136. doi: 10.1371/journal.pgph.0002136 (PMC10802966; doi:10.1371/journal.pgph.0002136)
Supplement: Attachment — Submitted filename: rebutal_letter_nov_22_23.pdf [file pgph.0002136.s015.pdf]

Journal Requirements:

1. Please review your reference list to ensure that it is complete and correct. If you have cited papers that have been retracted, please include the rationale for doing so in the manuscript text, or remove these references and replace them with relevant current references. Any changes to the reference list should be mentioned in the rebuttal letter that accompanies your revised manuscript. If you need to cite a retracted article, indicate the article's retracted status in the References list and also include a citation and full reference for the retraction notice.

We have reviewed our reference list to ensure that it is complete and correct. We have removed the papers that were preprints and replace them with the corresponding published manuscripts. No cited article was retracted.

Reviewer #1: The authors improved the article and now is clearer. Due to the large number of details, I understand that it is difficult to cover all or easy to miss aspects that readers would be interested.

The authors added in the new version very general references related to mathematical models that have been used for the COVID pandemic. However, since one specific aim of this work is to compare vaccine allocation strategies the references and discussion should be related to this specific aim.

We thank the reviewer for this comment. We have added additional references and discussion to the manuscript.

In the modeling contacts section, it says that the user must select the multiplier (between 0 and 1) related to the social contact. One represents full contact. Please add further detailed explanation for this. Many readers can be confused. There are 4 social contact matrices and a force of infection that depends on the values of these matrices.

We thank the reviewer for this comment. We have added a further detailed explanation for this, and we refer now the reader to the particular section of the supplement where the explicit formulas are given.

In the modeling initial conditions please mention how many values the user needs to provide. Please remark or comment all the difficulties that this aspect might have for a user. This point is crucial for the outcomes of the simulation.

We have added the minimal number of numbers that are needed for initial conditions in the model, and we have added a section in the discussion about this point being a limitation.

1- Please mention the assumed proportion of asymptomatic in this section or clarify that is also an input provided by the user.

Thanks for raising this point. We have added a sentence to this section clarifying this point and referring the reader to Table S2, in the SM where provides the assumed proportion of asymptomatic infections are provided.

Page 7. "If the vaccine is used as a booster, the user also provides the age and previously vaccinated groups where the booster will be applied." This phrase is not clear.

We have rewritten this section to make it more clear to the reader. We thank the reviewer for raising this point.

Please explain how the allocation of strategies per day is incorporated in the model. What type of ODE model it is. These small details can help readers to grasp the methodology and the suitability for each region. Looking and explaining Eq. (4) can help.

Thank you for this comment. We have added a section in the methods entitled "Vaccine allocation implementation" explaining how the model does this.

Page 8. "However, we assume that two infection events act as a primary series vaccination. "Please add a brief explanation and/or one reference for this assumption.

We have added two references for this assumption. There is a large body of literature indicating that infection events induce protection. We however also recognize that a single infection event might be enough to induce similar protection as a primary series. We added this as a limitation in the discussion.

In the discussion section I think is fair to add further limitations. For instance, the user needs a good knowledge of the parameters of the model. In addition, the user needs to have knowledge of the initial conditions (please add how many values are needed as an input). Also, the authors should emphasize or remark that the number of strategies is quite large and for a user might be impossible to check all the potential strategies. The examples provide few strategies. I understand that including more examples could be demanding, but mentioning this can help the readers.

We agree, we have expanded the limitation section of the discussion considerably.

The aim of the article is worthy and important. The aims are very challenging due to all the factors that affect the outcomes of the simulations.

We thank the reviewer for their assessment of our work.

In the supplementary part please add the Ro as explicit as possible. This helps the readers.

Thank you for the comment. We have added a few sentences explaining what R0 is and how it is used in the model.

Page 36. Typo experience.

Thank you for catching this typo!
